# Effects of using an abdominal simulator to develop palpatory competencies in 3rd year medical students

**DOI:** 10.1186/s12909-022-03126-y

**Published:** 2022-01-26

**Authors:** Robert M. Hamm, David M. Kelley, Jose A. Medina, Noreen S. Syed, Geraint A. Harris, Frank J. Papa

**Affiliations:** 1 Department of Family and Preventive Medicine, University of Oklahoma Health Sciences Center, 900 NE 10th St., Oklahoma City, OK 73104 USA; 2 Physician Associate Program, University of Oklahoma Health Sciences Center, Oklahoma City, OK USA; 3 Great Plains Family Medicine Residency Program, Oklahoma City, OK USA; 4 Texas College of Osteopathic Medicine, University of North Texas, Fort Worth, TX USA; 5 ACDET, Inc., Fort Worth, TX USA

**Keywords:** Abdominal examination, Diagnosis, Medical students, Palpation, Physical examination, Simulation

## Abstract

**Background:**

Medical school faculty are hard pressed to provide clerkship students with sufficient opportunity to develop and practice their capacity to perform a competent clinical examination, including the palpatory examination of the abdomen. We evaluated the impact of training with an abdominal simulator, AbSim, designed to monitor the depth, location, and thoroughness of their palpation and to provide concurrent and summative feedback regarding their performance.

**Methods:**

All third-year medical students were given the opportunity to develop their palpatory skills with the AbSim simulator during the family medicine rotation. The performance of those who studied with the simulator was measured by its sensors, before and after a training session that included visual feedback regarding the depth and coverage of the student’s manual pressure. Additionally, all students reported their confidence in their evolving abdominal palpation skills at the beginning and end of the rotation.

**Results:**

119 (86.9%) of 137 students filled out the initial questionnaire, and 73 (61.3%) studied with the abdominal simulator. The training produced a highly significant improvement in their overall performance (4 measures, p’s < 0.001). Pre-training performance (depth calibration and thoroughness of coverage) was not related to the number of months of previous clinical rotations nor to previous internal medicine or surgery rotations. There was little relation between students’ confidence in their abdominal examination skills and objective measures of their palpatory performance; however, students who chose the training started with less confidence, and became more confident after training.

**Conclusions:**

Guided abdominal simulator practice increased medical students’ capacity to perform an abdominal examination with more appropriate depth and thoroughness of palpation. Interpretation of changes in confidence are uncertain, because confidence was unrelated to objectively measured performance. However, students with low initial confidence in their abdominal examination seemed to be more likely to choose to study with the abdominal simulator.

**Supplementary Information:**

The online version contains supplementary material available at 10.1186/s12909-022-03126-y.

## Introduction

We report a study assessing the use of an abdominal simulator to give 3rd year medical students practice experience with the palpation of the abdomen, an essential skill for rapidly evaluating and accurately diagnosing abdominal pain [1, 2]. A competent palpatory exam builds on a basic understanding of abdominal anatomy and pathology, knowledge which can be acquired from text and graphics, yet it also has sensory-motor components. As it involves sensory motor skills, we may assume it is best learned through actually doing the physical movements, rather than simply watching [3]; and through repeated practice with appropriate feedback [4]. As a review of 32 studies showed, the more hours spent on a clinical skill the more it is mastered [5]. However, palpation of patients’ abdomens evokes pain, even when executed by skilled clinicians, and so patients and teachers are hesitant to permit repeated exploration by learners. Consequently, students’ initial development of abdominal palpation skills is often derived from palpation of one’s fellow students or “standardized patient” actors [6]; they can tolerate repeated palpations, but their abdomens do not accurately demonstrate how various abnormalities feel, and their acting may not accurately reflect patients’ responses. Well-designed abdominal simulators could potentially fill the gap [7], as they could be designed to approximate the feel and location of various abnormalities and some aspects of the behavior of patients with sensitive abdomens, without incurring discomfort in actual patients.

There has been a rapid growth in the number and variety of simulators used in medical education. These can range from very simple devices that give practice in specific tasks (e.g., listening to lungs and hearts, stitching wounds, making surgical incisions, or performing injections) to highly sophisticated computer controlled manikins that can provide individuals or teams with realistic experiences, extended through time; opportunities to perform a variety of complex manual procedures or to manage cognitively demanding medical emergencies [8, 9]. Compared with traditional exposure to patients in clinical rotations, deliberate practice using simulation often provides a superior learning environment [10]. Twelve simulator features and best practices for integrating simulation into medical education have been identified [11]. A simulator that focusses on palpation was described by Anders et al. [12], whose device measures manual pressure intensity and rate of applying it for the chiropractor’s clinical task of identifying and treating myofascial pain.

There are many reports at conferences and in the literature describing abdominal simulators. The range of their technical sophistication varies, from a manikin which the teacher sets up for the student by inserting rubber physical organs into the cavity [13, 14] to ones with computerized control of a menu of abdominal abnormalities and concurrent and summative feedback of student performance (such as the device used in the current research).

Unfortunately, while companies around the world (e.g., Laerdal in Norway, General Doctor in China, Oniko in the Ukraine) advertise abdominal palpation manikins, evidence of their capacity to objectively measure and improve students’ physical examination capabilities has not been described in the literature. Further, there has been little research evaluating simulation’s contribution to mastery of the physical examination of the abdomen. In a large review [7] the only mention of learning to examine the abdomen was as a control condition. As of 2012, there had yet to be any comparative effectiveness studies comparing an abdominal simulator with a specified alternative teaching method [15]. Ribeiro [16] reviewed the theory and practice of constructing devices that provide accurate simulation of the haptic features [17] needed for training of palpation or surgical procedures, noting that “most of the studies consider only one point of contact, which can limit the simulation realism,” ([16], page 1) but did not include any assessments of devices designed specifically for the palpation of the abdomen.

Despite exhortations that the evaluation of educational interventions should use good measurements [18–20] that are related to relevant theories of the acquisition of knowledge and skill [4, 9], it has been observed that much research assessing methods of medical education is methodologically weak [21]. A 2011 review of simulation in medical education found only 14 papers comparing simulation-based education with traditional methods [10].

In the present study, the educational intervention focusses on the execution of one particular component of the skill of diagnosing abdominal illness, the palpation of the abdomen. It is measured with the lowest two of Kirkpatrick’s 4 levels [22]: students’ self-assessed evaluation of the learning experience (level 1), and objective measures of performance (level 2). Thus this study does not address students’ clinical behavior nor patient outcomes, measurements which would be infeasible for routine medical school instruction and inappropriate for training that is focused on only a particular subcomponent of a skill.

AbSim, the abdominal simulator used in this study, was designed to train the basic elements of the physical examination of the abdomen. It has a realistic-feeling torso with the capability to simulate a number of abnormalities (e.g., appendicitis) and patient responses to palpation (vocal, abdominal wall guarding), and to provide students with feedback regarding their technique (depth and location of palpation with respect to likely location of organs). In the present study we made use of its ability to provide students with feedback regarding the depth, location, and thoroughness of their palpation of a realistic feeling abdomen, without any exposure to the feel of abdominal abnormalities or to patient behavior during examination of a tender organ.

The main objective of the study was to measure the effect of the simulator training on objective measures of the depth and thoroughness of the students’ palpatory examination of the abdomen. A secondary objective was to measure the training’s effect on students’ confidence in their ability to do the abdominal examination. Because the treatment was not randomized, pre-training measures were used as the comparison. Further, because students chose whether to participate, measures of previous clinical training experience (months of previous 3rd year clerkships and particular clerkships that teach physical examination) as well as students’ initial confidence were controlled for in multivariate analyses of the effect of training. A third objective was to assess students’ attitudes toward training with an abdominal simulator, and a fourth was to use the AbSim measurements to characterize how student performance of the palpatory examination improves during their first clerkship year.

## Methods

### Participants

All 3rd year medical students at this public allopathic medical school are required to take a 4-week rotation in family medicine. They were asked to volunteer for the study during class on the first day of the rotation.

### Abdominal simulator

The study used the AbSim abdominal simulator,^1^ a model with a frame approximately 56 cm long, 35 cm wide, and 27 cm tall, covered with anatomically correct silicon skin, with visible umbilicus and palpable ribs and pelvis defining the bounds of the abdomen. Underneath the visible layer are a layer of spongy padding, a sensor pad, and a taut silicon sheet that supports the sensor pad.^2^ The attached computer has a program with which the researcher can display a schematic outline of an abdomen showing the location of pressure applied by the learner and detected by the sensor pad, in relation to the locations of key abdominal organs.

The researcher used the simulator to provide training directed at developing sensory and motor skills representing appropriate: 1) depth and 2) thoroughness of the student’s performance of an abdominal examination. The simulator’s various multi-modal formative feedback mechanisms (tactile, visual and vocal feedback) were used both to reinforce appropriate palpatory depth and thoroughness, and to call attention to the need for remediation.

**Fig. 1:**
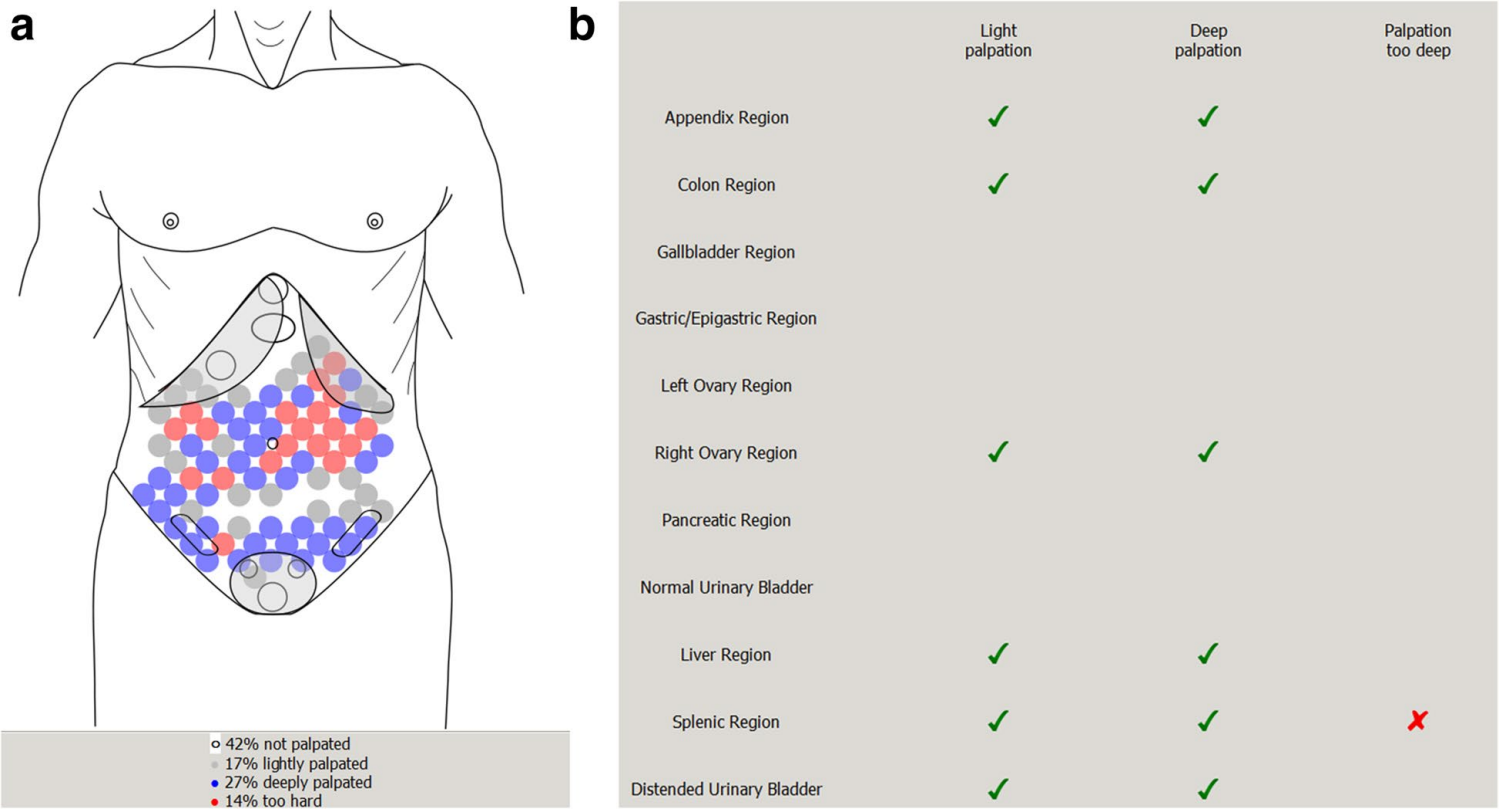
Display of palpation exam shown to student, showing deepest palpation detected at each location (**a**) and at the location of each of 11 organs (**b**)

Three forms of feedback to the student were utilized in this study: 1) display of the pressure the student is currently exerting wherever it is sensed, coded as light (gray), deep (blue), or too deep (red); (2) cumulative display of maximum pressure detected at each location during an exam, superimposed on a sketch of the abdomen that includes outlines of organs in their typical locations (Fig. 1a); (3) list of organs with check marks indicating whether light, deep, or too-deep palpation was detected at the organ’s location (Fig. 1b). The boundaries between no, light, deep, and too-deep palpation were calibrated based on the clinical experience of multiple physicians both locally and at various conferences (see Appendix 2 in Supplementary Materials).

### Questionnaires

At the beginning (Monday of first week) and end (Wednesday of last week) of the 4-week clerkship, all participating students filled out a questionnaire addressing their confidence regarding examining the abdomen and their preferences for various modes of instruction (see Supplement, Appendix 1). Confidence was measured using the average of seven ratings of one’s “competence” performing various aspects of the abdominal examination. The questionnaire at the end of the clerkship also asked 5 questions regarding their evaluation of studying with an abdominal simulator, how many real patient abdomens they had palpated during the month, and whether they had previously done their 8-week clerkships in surgery and internal medicine (during which the abdominal exam is also commonly taught). We have used this questionnaire in similar unpublished studies of the abdominal simulator since we wrote it in 2015.

### Measurement

The simulator’s software allowed measurements of the depth and thoroughness of the subjects’ palpatory efforts, both prior to and following training. It stored screenshots documenting each student’s 1) depth of palpatory efforts (characterized as none, light, deep or too deep, extracted from Fig. 1a for each student’s two observations; data summarized in Fig. 3), and the thoroughness of their palpatory effort (measured in terms of the specific areas of the abdomen palpated and not palpated; summarized in Fig. 1b for each student, for areas outlined in Fig. 4; data shown in Figs. 5 and 6). These measurements were taken twice before and twice after the training, once with instructions to demonstrate one’s light palpatory technique, and again with instructions to demonstrate deep palpation. Additionally, during the simulator’s training sequence the software showed the student a “baseline” summary of the student’s performance on another “light, and then deep” examination, and subsequent to the training that process was repeated as a “comparison” summary. These summaries are not stored by the computer, but while the student reviewed them the researcher saved a screenshot.

All six of these displays were analyzed by calculating the extent to which the student explored the 11 organs at the requested depth. The baseline and comparison reports also showed the thoroughness of the subjects’ palpatory efforts (i.e., the percentage of the abdomen that was palpated or not palpated) and the percent of the total abdomen palpated at each depth. The scores on the initial three observations were used to assess the impact of students’ previous clerkship experiences as the third year passed. The comparison of these scores from before and after the training identifies changes in palpatory technique (in both thoroughness and depth) resulting from their use of the simulator, and provides measures of the effect of Absim training.

In more detail: To measure the quality of students’ abdominal examination when instructed to palpate *lightly*, we calculated summary scores from the visual report of the light exam for each of 11 organs, for each region of the abdomen, and overall. This score gives most credit for a palpation measured as light, less for deep palpation, even less for too-deep palpation, and 0 for no palpation. A similar procedure applied to the report of the *deep* exam yields a score of how well calibrated the student is when instructed to palpate deeply. Formulas are in Fig. 5a and b. The deep exam scoring is also applied to the baseline and comparison records produced as part of the tutorial; though these assessments required both light palpation and deep palpation, the computer reports only the deepest palpation sensed during the entire exam. Finally, the simulator’s tutorial record also reports the proportion of the area of the abdomen (including areas outside the 11 specified organ locations) that were palpated lightly, deeply, too deeply, or not at all.

#### Procedure

During the clerkship’s first day, a researcher explained the study, sought informed consent for participation, administered the initial questionnaire, and offered signup sheets for students to schedule individual sessions studying the abdominal simulator with a researcher. It was stated that even if they did not plan to study with the simulator, their questionnaires were of interest. Reminders were sent using the campus email and calendar system (Microsoft Outlook). For participants who did not schedule a session because they did not yet know their clinic schedule, one email message was sent offering open slots once they had a better idea of their availability. Effort was made to schedule the session during the first week, though occasionally it was necessary to schedule during the second week.

To start the individual sessions, the researcher oriented the student to the simulator (see Appendix 2), and explained that there would be an assessment, then training, then another assessment. In the pre-training assessment, the subjects were requested to perform three separate palpatory examinations of the abdomen: first palpating only lightly, then palpating only deeply, and third palpating both lightly and deeply. During these assessments the students were given no visual feedback regarding the depth or location of their palpatory efforts via the simulator’s monitor, until after the third examination, when the monitor was turned so they could see its record, as the baseline for the upcoming training.

The training sequence consisted of four phases. 1) Inspection of the baseline summary (e.g., Fig. 1a and b) which shows a graph of the location and depth of their palpations, and a list of the areas of the abdomen where key organs are located (appendix, gallbladder, ovaries, etc.) and the maximum depth with which they had palpated each area. 2) Training about the appropriate depth of palpation. The student palpated the abdomen while observing the schematic torso outline without organ locations, as the current depth and location of their palpation was displayed on the screen (colored dots indicating light, deep, and too deep palpation). This training was intended to enable the students to calibrate the depth of their palpatory efforts to those criteria established by the investigators, enabling them to recognize when their palpatory efforts are light, deep and too deep. 3) Training about the appropriate thoroughness of palpation. This training consisted of the student palpating the abdomen while observing a schematic torso on the monitor, which this time displayed the specific locations of the various organs likely to present as common and/or important abdominal disorders. The monitor provided real time, visual feedback revealing which areas of the abdomen (and the underlying organs) they had palpated so far. This cumulative display of the deepest pressure they had explored with at each location was intended to reinforce the performance of a thorough palpatory examination of the abdomen. 4) Performance of a complete “comparison” palpation, both light and deep, with monitor screen turned away, and subsequently viewing their coverage and depth, comparing how they had done before versus after the training, and discussing with the researcher what was palpated and what was missed. Students were allowed to take as long as they wished on each phase of the training. The training time was not recorded, but most of the students completed the entire session within a half hour.

After the training, for assessment the student repeated the separate light and deep abdominal examinations, with each recorded separately and with no feedback. Finally, the student was reminded of the importance of filling out the questionnaire at the end of the clerkship.

During the fourth and final week, participating students were given the second questionnaire by the clerkship administrative assistant (with the statement that completing it was important even if one had not had a session with the simulator). Participants who had not filled it out were sent a copy (Microsoft Word document) by email with encouragement to complete it and send it back.

#### Data analysis

Data were gathered at 3 time points during the 4-week clerkship (first day questionnaire; first week AbSim training; last week questionnaire). Analysis addresses the prediction of measures at each time point from preexisting measures. Thus, we use demographics and previous clerkship experience to predict initial confidence (self assessment of competence) in one’s abdominal examination skills; demographics and initial confidence to predict participation with the simulator and pre-training performance (palpation depth and coverage); demographics, initial confidence, and initial performance to predict post-training performance; and all the above to predict end of rotation confidence and attitudes regarding training with an abdominal simulator. Because not all students used the simulator, two separate analyses are required for some of these models, one with all students but excluding performance data, and one including performance data that includes only students who did the tutorial.

The questions addressed include: Does previous 3rd year training improve students’ initial confidence and competence, and whether they are interested in spending time training with an abdominal simulator? Does the training improve competence and confidence? Do the students think that the training was worth the time?

For each prediction three levels of relation were done using current versions of IBM SPSS; pairwise relations between each predictor and the dependent variable (correlations measured with Pearson’s r, differences compared with t-tests for continuous variables and Chi-square test for using AbSim or not); multivariate analyses (linear and logistic regression); and structural equation modeling. Summary results are presented in the main paper, organized by interest and importance; the remaining results are in the Supplementary Appendices, which are organized according to the framework described above.

## Results

### Participants

**Fig. 2:**
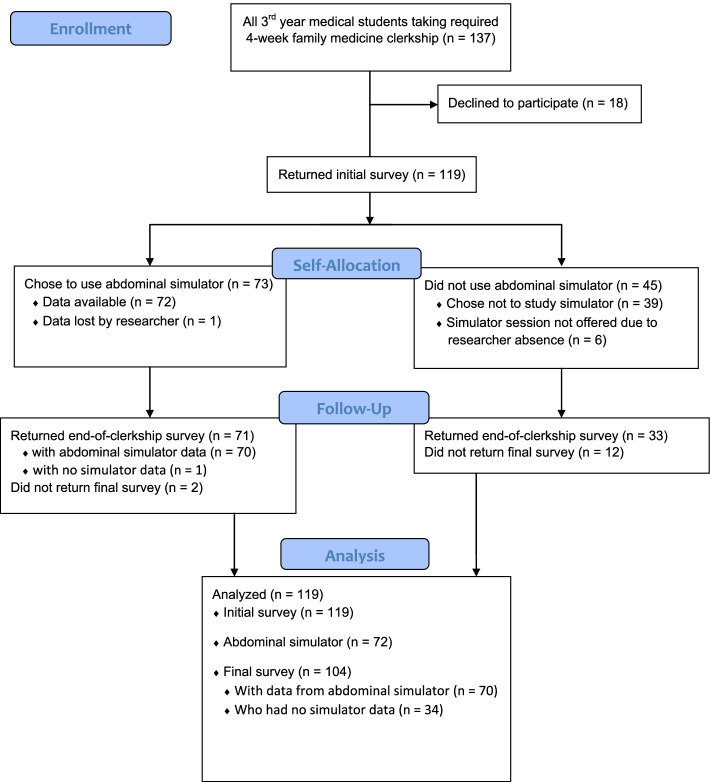
Flow diagram of student participation in surveys and abdominal simulation training

One hundred thirty-seven third year medical students were enrolled in the twelve family medicine clerkship blocks over the academic year (July 2017 to June 2018); 119 (86.9%) of these agreed to participate in the study and filled out the initial questionnaire (see Fig. 2). Of these participants, 73 (61.3%) completed a session using the abdominal simulator (all data for one of these was lost, as well as some data for 10 other students), and 104 (87.3%) filled out the final questionnaire. There was no trend over the year in the proportion of students enrolling in the study to complete the initial questionnaire, *r* = − 0.05 (excluding the final month, June, when the students knew no simulation training would be provided due to researcher absence), and a nonsignificant decrease in the proportion of participants who completed the final questionnaire (correlation with clerkship month, *r* = − 0.31). However, the proportion of participants who arranged to study with the abdominal simulator declined greatly over time: the 3-month averages were 81% for July–September, then 73, 61%, and finally 30% for April – May, with a − 0.70 correlation between participation rate and month (*p* < .01). (See also Supplement, Appendix 3.)

#### Performance: overall changes in depth calibration and coverage, in students’ exploration of abdomen

**Fig. 3:**
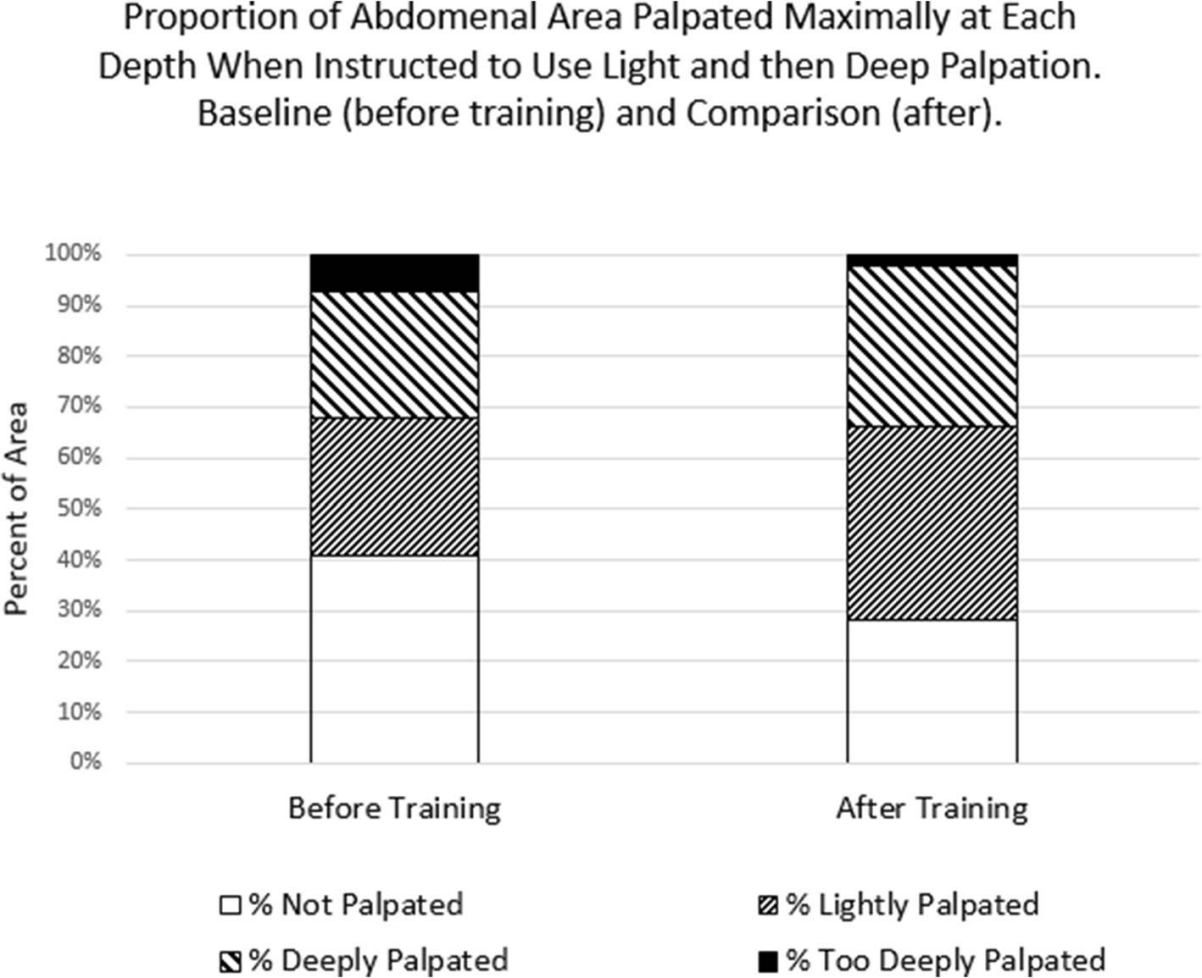
Comparison of proportions of the abdomen palpated at each depth, before and after the palpation tutorial

The baseline and comparison reports provided by the simulator in its tutorial mode included an estimate of the proportion of the abdominal area that had been palpated lightly, deeply, too deeply, or not at all (Fig. 1a). As the student had been instructed to examine the abdomen using both a light and a deep touch, a deep palpation should have been registered at each location. After training, the average proportion deeply palpated increased (t = 3.7, df = 60, Cohen’s d = 0.48, *p* < 0.001), though it was still less than 50% of the abdominal area (Fig. 3; data are tabulated in Appendix 4). The proportion lightly palpated also increased (t = 6.8, d = 0.88), while there was much less “too deep” palpation (t = 6.7, d = 0.86). The students learned to perform more thorough exams, because the proportion not palpated decreased by almost a third (t = 9.7, d = 1.25).

#### Changes in the palpation of various organs

**Fig. 4:**
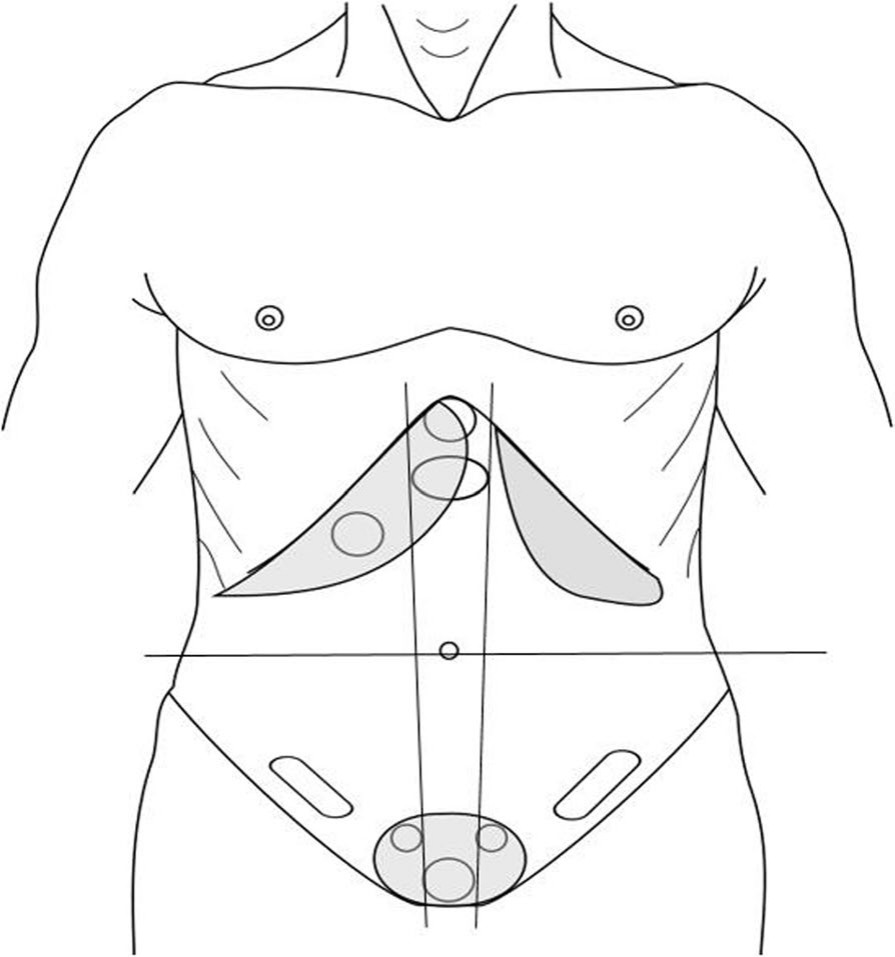
Torso with organ locations identified. Lines (not shown to students during assessments) define left, middle, and right parts of upper and lower abdomen

**Fig. 5:**
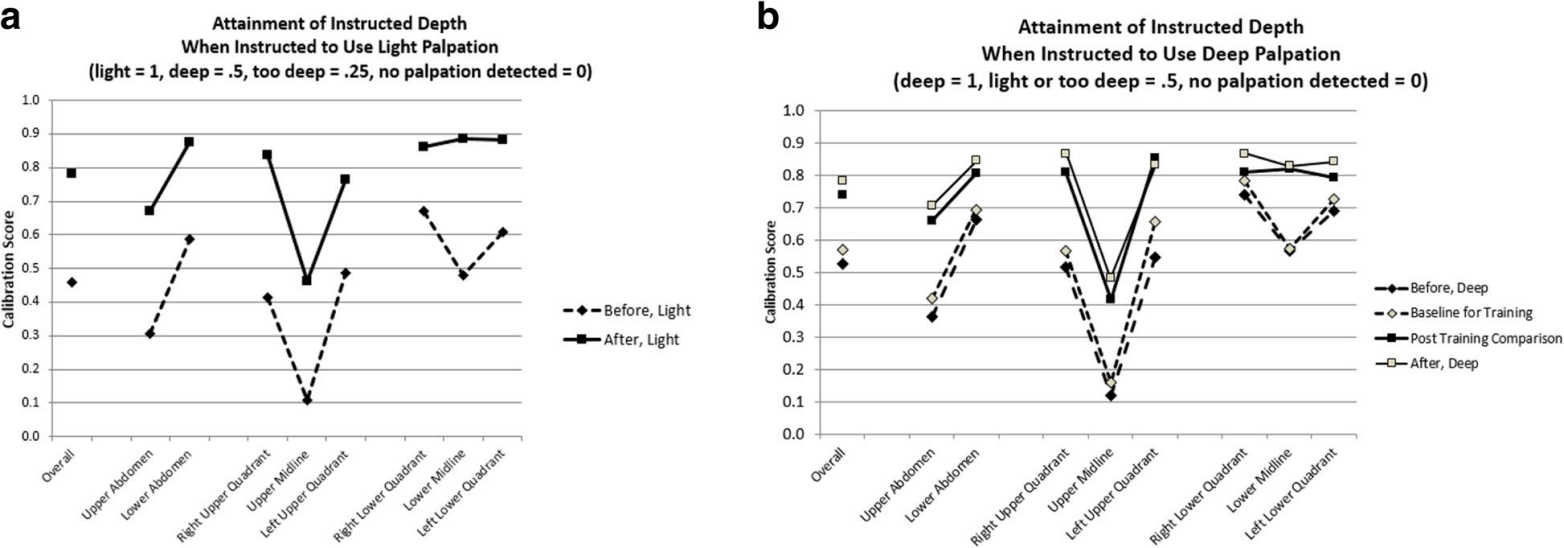
Scores measuring light (**a**) and deep (**b**) palpatory examination, for when instructed to use light or deep technique, before and after training

From the record of the depth of palpation over each organ (Fig. 1b) we computed the student’s average light and deep scores in each zone, as defined in Fig. 4. At every level of aggregation, there was general improvement in the students’ ability to demonstrate an appropriate (i.e., expert-calibrated) level of light palpation in each (Fig. 5a and Appendix 4; each t-test significant with *p* < 0.001, *N* = 72 students; overall average score improved from 0.45 to 0.78, d = 1.40). The ability to demonstrate an appropriate deep level of palpation also improved (Fig. 5b; each t-test significant at *p* < 0.001, *N* = 72, except for the organs located in the left lower abdomen (left ovary, colon), *p* = 0.04, and the upper middle abdomen (gastric/epigastric region), *p* = 0.34; overall scores had effects of d = 0.88 (baseline versus comparison) and 1.37 (deep instructions, before versus after).

#### Use of the simulator’s recording capabilities to assess students’ performance before simulator training

Presumably third year medical students are improving their physical exam skills as they rotate through various clerkships. The simulator’s pre-training metrics should reveal changes in the depth and thoroughness of their palpatory abdominal examination over time, as influenced by previous rotations, independent of any simulator training. There was no statistically significant change over the year in the proportion of the abdomen palpated at each depth (correlations with month ranged from *r* = − 0.222 for % not palpated (*p* = 0.08) to *r* = 0.183 for deep palpation (*p* = 0.16)).

Besides family medicine, the surgery and internal medicine clerkships probably offer students the most instruction and experience with the physical examination of the abdomen, with less in the obstetrics & gynecology and pediatrics clerkships. As the year progressed, it became more likely the students had experienced each specific clerkship when they appeared for the abdominal simulator training session. Having completed surgery or internal medicine seemed to produce slight improvements on the various abdominal simulator measures prior to this study’s training, but the differences were not statistically significant (Appendix 6b).

**Fig. 6:**
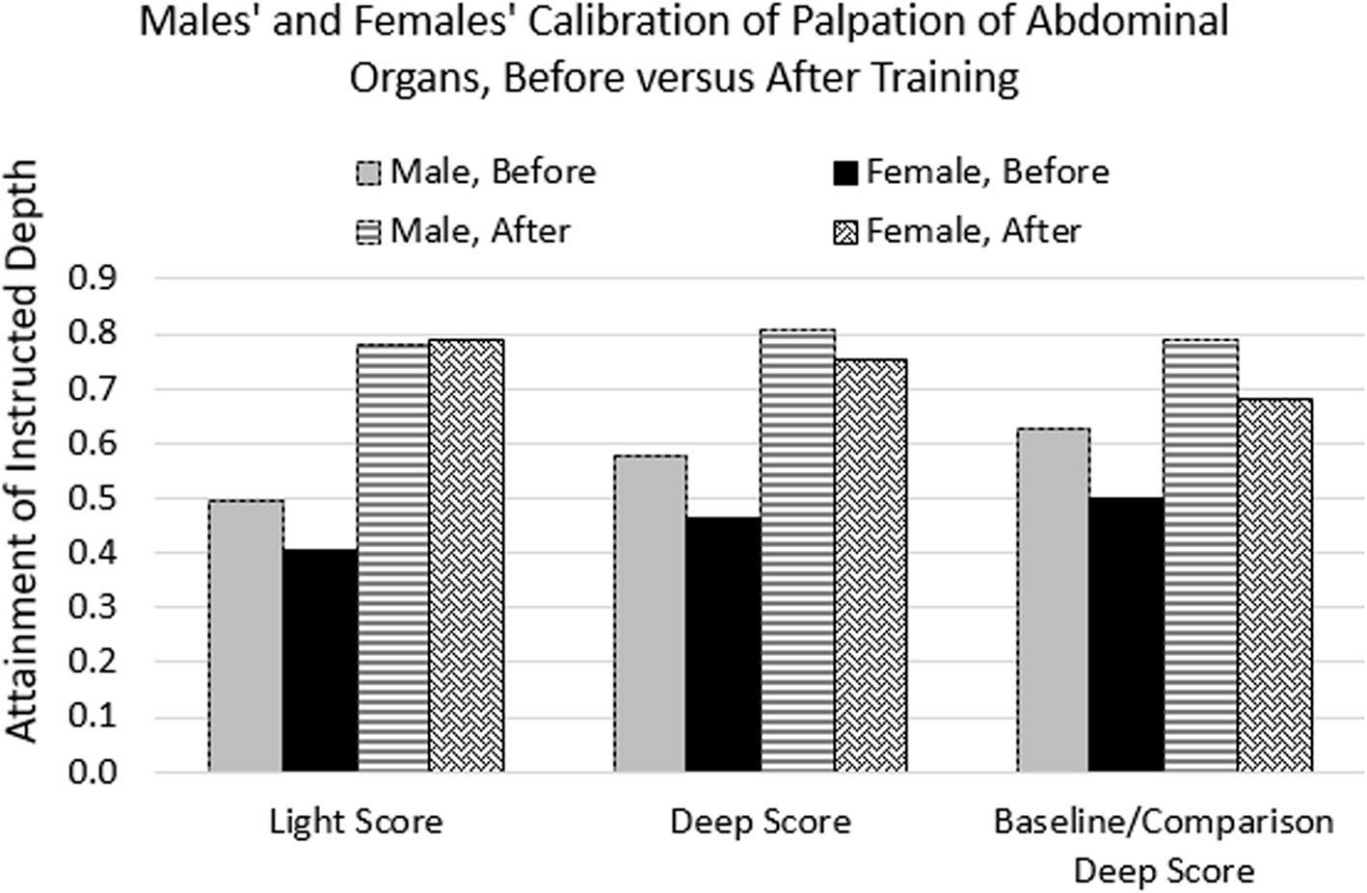
Comparison of light and deep calibration scores for male and female students, before and after simulator training

*Gender differences* in depth of palpation and thoroughness. Though not planned, we looked at possible differences in the depth of the palpatory efforts of males (*N* = 41) and females (*N* = 31). Before training, males tended to demonstrate appropriate (i.e., expert calibrated) levels of both light and deep palpation more than females (Fig. 6 and Appendix 4: light ideal: difference = 9%, *p* = 0.06; deep ideal: difference = 13%, *p* = 0.006; deep ideal on training Baseline, difference = 13%, *p* = 0.007). These gender differences were somewhat reduced but still significant after training (light ideal: difference = − 1%, *p* = 0.72; deep ideal: difference = 5%, *p* = 0.04; deep ideal on post training Comparison, difference = 11%, *p* = 0.002). There was a similar pattern, of initial gender differences reduced by simulator training, in the thoroughness of the abdominal exams. On the baseline examination before training, on average the men palpated a greater percentage of the abdomen than the women (61% vs 55%, F = 3.99, *p* = 0.05). On the post-training comparison exam, the difference (72% vs 70%) was not statistically significant.

#### Relations among initial confidence, studying with the abdominal simulator, and final competence

The confidence of those studied with AbSim improved more, from the beginning to the end of the clerkship, than that of the students who did not (F = 8.1 for the difference in improvement, *p* = 0.005, Cohen’s $\hat{f}$ = 0.28). However, the multivariate analyses in Appendix 6 suggest that student self selection into the group that trained with the simulator confounds the comparison of the self confidence of those who did and did not train. For example, as the third year passed, students reported more confidence (at the beginning of the family medicine clerkship) in their abdominal examination skills and fewer chose to study with the simulator. Additionally, within each month, those who reported having less confidence were more likely to study with the simulator. Studying with the simulator increased students’ confidence, from the beginning to the end of their clerkship, yet this served only to catch them up with the confidence of those who did not study with the simulator. Structural equation models can address all these variables simultaneously. The structural equation analysis reported in Supplemental Appendix 7a shows that all three links delineated here are statistically significant, consistent with the suggestion: High initial confidence and abdominal simulator training each predict higher confidence at the end of the rotation, and higher initial confidence predicts that the students will NOT study with the simulator.

#### Student evaluation of simulator training

Do students who have studied with the abdominal simulator state a more positive evaluation and attitude about it than students who did not study with it, on the final questionnaire? Among the students who worked with the abdominal simulator, most felt they had learned from it, though they qualified the statement. Thus, 19 (31%) said they learned a lot; 19 (31%) said they learned a little but would need to use it more to really benefit; 20 (33%) said they learned a little and it was about all they would ever learn from such a device; and 3 (5%) said they learned nothing new. This question was not relevant for those who did not study with the abdominal simulator.

Asked whether they’d want to study with an abdominal simulator without an instructor, 93.4% of 61 students who had studied with the AbSim said “yes,” compared to 84.4% of 32 students who did not study with it (NS, Chi-Square (df = 1) = 1.974, *p* = 0.16). Asked whether classroom demonstrations with an abdominal simulator, without the opportunity to palpate it, would be useful, 50.8% of those who had used the AbSim and 59.4% of those who had not used it said “yes” (NS). Asked whether they would want to have guidance using an abdominal simulator, or to use it independently, 63.9% of the AbSim studiers and 65.5% of the non-studiers said they’d prefer guidance (NS). 77.0% of the AbSim studiers and 63.3% of the non-studiers would recommend integrating the abdominal simulator into the Family Medicine clerkship curriculum (NS).

Students were asked to rank order 9 modalities for learning about examining the abdomen at both the beginning and the end of the clerkship. There was little change in the relative preference for studying individually, or as a group, with an abdominal simulator, either among those who studied with the AbSim or those who did not. The most preferred mode was a standardized patient, then a real patient, and then individual study with an abdominal simulator which was tied with palpating a fellow student. The less preferred modalities were instructor demonstration, video tutorial, lecture with powerpoint, and finally reading.

## Discussion

We have observed that for instruction in the palpatory examination of the abdomen, there is little focused, well-defined instruction with objective assessment. We described a simulation trainer, and in a study with before–after measurements operationalizing depth of palpation and thoroughness of coverage we showed that the training produces improvements in both performance as measured using the simulator and confidence as measured by student self report of competence.

The literature on training medical students in the physical exam of the skin shows a similar state of development as that on abdominal palpation. There are papers demonstrating that students receive little explicit dermatological training, and that students have varying objectively-measured performance and self confidence in their skills [23, 24]. While a variety of papers address training students to avoid cognitive errors in diagnosis [25, 26], fewer assess specific programs providing instruction in particular techniques for a thorough dermatological examination, other than providing more exposure to videos [27] or dermatology teaching time during a clerkship [28]. Perhaps the best methodology was exhibited in a study by Choi et al. [29] in which students in groups of 3 or 4 were led by an instructor in a structured process of describing example skin lesions, diagnosing them with feedback, and reflecting on the process; this led to improved performance on a later test (d = 1.5 compared to no intervention, 1.2 compared to a lecture). However, this was in-person training without a simulator.

Training in endoscopy is further advanced, both in the technical sophistication of the simulators and in the research methods for evaluating the effects of the training. The components of adequate colonoscopic exam have been delineated [30], and a number of studies evaluating colonoscopic exam performance with various specific measures have been reported (reviewed in [31]; simulator education studies reviewed in [32]). A study by Kruglikova et al. [33] used an approach very similar to that of the present study. Twenty-two students did 15 repetitions of one particular task from the curriculum embodied in a simulator’s training program. Performance was assessed using the simulator’s measurement capabilities (percentage of mucosa visualized, time to reach caecum, lack of perforations), which had been calibrated by experienced colonoscopists, and two of 10 comparisons were statistically significant at *p* = 0.03.

Our study of the impact of using an abdominal simulator to train 3rd year medical students in key palpatory aspects of the physical examination of the abdomen showed that the training produced significant improvements in the calibration of the depth of the medical students’ abdominal palpation, and in the thoroughness of their examination of the abdominal organs. This study makes a contribution because no study has previously been published showing the effects of abdominal simulator training on these two skills. This improvement was larger than produced by the training and clinical experiences the students had had on their previous rotations. It had effects similar to the training provided by Choi et al. [29] in dermatology, and larger than those observed by Kruglikova et al. [33] in colonoscopy. In Choi et al’s study of the effect of an hour’s structured study of 10 lesion pictures with instructor feedback, students in the intervention group diagnosed cases better a week later on a test of the trained diseases, a large effect size (a difference of 1.5 standard deviations). In our study, the size of the effect of the training on confidence, compared to those who did not train, was Cohen’s $\hat{f}$ = 0.28. The effect of training on proportion of the abdomen palpated ranged from Cohen’s d = 0.48 to 1.25, and on the appropriate depth of palpation ranged from d = 0.88 to 1.40.

This study involved both main types of simulation-based research described by Cheng et al. [18]: “1) studies that assess the efficacy of simulation as a training methodology and (2) studies where simulation is used as an investigative methodology” (p. 1091). The AbSim simulator provided the training, and its measurements before and after are the source of the objective measurement of previously acquired competence and of changes in competence following simulator training, similar to a previous study of colonoscopy training [33].

The AbSim abdominal simulator used here has unique features: it a) simulates the feel of the abdomen; b) measures the student’s palpation, both position and depth; and c) provides these measurements as both concurrent and summary feedback for the student. To the best of our knowledge, it is the only commercially available abdominal simulation trainer that has these useful features, and that has software specifically designed to take the learner through a sequence of experiences that build up competence. Still it is not completely realistic. It is full of air, not liquid, so it does not make informative sounds when tapped. The resistance to a probe is provided by a taut rubber sheet, not by variously shaped organs suspended in liquid. Its “skin” is that of a slender person, so it does not provide experience palpating through various thicknesses of fat.

The training procedure includes 5 of the 12 features that McGaghie et al. [11] identified as promoting learning: “(i) feedback; (ii) deliberate practice; (iii) curriculum integration [in that the students may well need it in their current clinical placement]; … (v) simulation fidelity; … (xi) instructor [researcher] training.” It can be viewed as a way to support the development of higher levels of performance through supervised practice of each part of a skill in isolation and subsequent reintegration with the execution of the whole skill [34, 35].

To address Bewley and O’Neil’s (2013) observation that much research assessing methods of medical education is methodologically weak, we aimed for: precise definition of the goals of the training in terms of particular subskills of competence; accurate and relevant measurements of the skill; effective training that provides the opportunity to practice the skill with concurrent as well as summative feedback; the best feasible sample; and an adequate design for evaluation of the effect of studying with the simulator.

The study’s sample was good in that over 85% of the students in the third-year medical school class participated by filling out the questionnaires at the beginning (and 75% at the end) of the family medicine clerkship month; and over 60% of these participants actually took the training, although the proportion doing so declined during the year. The observations were taken over the course of 11 months, so the results are applicable to students at all points in the educational calendar. However, practical considerations – the rights of research participants, and an already overcommitted curriculum – precluded requiring all students to participate. Hence study participation was voluntary, as was whether participants studied with the simulator. We did not randomize students to studying or not studying with the abdominal simulator, and we had no palpatory performance measures for the nonusers of the simulator. Further, to minimize burden (which could have reduced participation), no measurement using the simulator was obtained at the end of the month; so the only performance data were obtained in 1 hour-long session at the beginning of the month, before and immediately after the training.

For each individual studying with the simulator we had multiple measures before and after the training, and the measures were taken in fine grain (dots at locations) and summarized in multiple ways (over all, and for particular regions and particular organs, and with separate assessments of light and deep palpation technique). While we found highly statistically significant improvements in the quality of the palpation examination immediately after training -- including the thoroughness of the students’ coverage, and the calibration of their light and deep palpation, overall and at most regions of the abdomen – we do not know how long these effects would last.

An incidental observation was that on the average female students started off palpating more lightly than men (on the other hand men more often palpated “too deeply”), perhaps because on average they weigh less or are more careful about not hurting people. The differences between men and women were somewhat reduced by the training. If upon further study these observations should be confirmed, it would be appropriate for educators to anticipate a need for individualized guidance.

One particular location that almost all students failed to palpate, even after training, was the epigastric area, i.e., the esophageal/gastric junction in the upper middle abdomen. Perhaps this is exceptionally difficult to explore adequately in real patients as well, suggesting that instructors should give special attention to this area. However, it may be a feature of this particular simulator: perhaps the sensors are not as sensitive in that region, which is near the edge of the sensor pad.

The students provided their assessments of the training (those who chose to do it) and of the general concept of simulator training (all participants). Despite the substantial improvements in objectively measured performance, there was no difference between end-of-clerkship evaluations by the participants who did and did not experience the abdominal simulator training. We had expected a difference. One interpretation is that participants who did not get simulation training chose not to because of difficulty in scheduling it (e.g., from their off-campus clinical site) rather than because of disinterest. An alternative interpretation would be that the training made little impression 3 weeks later.

### Use of abdominal simulator to measure student performance

We asked whether the students’ previous clerkship rotations, especially surgery and internal medicine, produced better performance as measured by the abdominal simulator prior to the students’ training. Our results indicate that the initial measure of palpatory performance was essentially level throughout the year; there was little measurable improvement due to having rotated through specialties in which abdominal palpation is necessary, nor due to the cumulative total of all previous clerkships. We had expected that prior experience would improve student competence and that it would be reflected in the simulator’s measures of their performance.

Assuming AbSim’s measures are valid, what does this finding imply? Perhaps students acquire accurate palpatory calibration on each clerkship and then rapidly lose it when it is not daily reinforced. Perhaps depth of palpation and through coverage of the abdomen are little emphasized elsewhere, because it is not viewed as the primary skill needed in the physical examination of real patient abdomens. Medical students may hear about these aspects of the physical examination of the abdomen on third year rotations, but not have much opportunity to practice. There was a large range in the amount of previous abdominal palpation experience students anecdotally reported to the researcher, depending on the particular clinics and physicians they shadowed. On the final questionnaire in this study they also reported a wide range of chances to palpate patient abdomens on the family medicine clerkship. A few students who exhibited clear competence, as observed by the researcher and measured by the simulator, explained that they had previously worked as emergency medical technicians or nurses. This emphasizes the important role of having the opportunity to practice on numerous patients, presumably with feedback.

What is proven by the increase in student performance after training, as measured by the abdominal simulator’s measurements, which naturally are linked tightly to the particular calibration standards as embodied on the simulator? At minimum, it indicates the students learned to temporarily adjust their palpation of the simulator in accord with the instructions – indeed it may be a very accurate measure of this particular subcomponent of abdominal examination competence. But it does not necessarily prove they have learned this skill permanently, nor does it prove competence at examining the wide range of body types, nor the broad range of examination skills taught by other physicians. However, the fact that the very experienced students did well on the simulator’s measures suggests it captures an essential part of an integrated competence. This implies that most medical students are not mastering the integrated skill of the physical exploration of the abdomen during their third-year rotations. Many of the students reported that they had not previously been guided to focus in such detail upon their palpatory technique, and so they paid close attention to the simulator’s feedback on how they were doing.

Is there value in having the simulator train students to perform the palpatory exam to a very particular standard – to calibrate to the level set by one set of experts – when patient body types and complaints may require a different range of pressures and techniques? We’d argue that there is benefit learning to execute a skill in a particular way, because one becomes aware of the range of possibilities. Training to a particular standard does not stop the physician from recalibrating when the situation demands it; rather it facilitates the ability to do so.

In conclusion, though we don’t know for sure if the simulator’s assessment of competence is generally valid, other than for immediate confirmation that the students adjust their calibration as the simulator tells them to do, there is plenty of indirect evidence to think it is. Presumably they learn how it is possible to calibrate, and they are made aware of depth and coverage of palpation as important aspects of their palpatory exam, whether or not the particular depth and coverage to which they were trained here is the best one in general.

### Limitations

An ideal study for assessing training with a simulator would be able to tell us the cost per long term stable improvement in competence, as compared to the costs and improvements of alternative training methods. Costs of training as done in this study would include buying or leasing the simulator and the time of the researcher who guided the student through the procedure. We did not test whether unsupervised students following only the instructions of the tutorial program would have the motivation to repeat the examination multiple times sufficient to improve their skills. Although we showed the training produced improvement between the pre and immediate post training measures of the particular subskills – depth and coverage – it is just part of the total skill of the physical examination of the abdomen, and other subskills merit supervised practice as well.

A major weakness, as we have acknowledged, is the non-randomized nature of the study, and the fact that even among study participants we had measures only from those who chose to arrange a session with the abdominal simulator. Thus we have competence measures only for those who were interesting in getting the training. We cannot compare the competence of participants who studied using the simulator and those who did not, nor with non-participants (who did not even fill out the questionnaires). Hence a possibility that our non-randomized non-comparative design cannot discount is that only the least competent students chose to study, as self-remediation. The fact that fewer participants opted to spend the hour with the simulator as the year went by is consistent with that alternative theory. It is less of a commitment for a program to provide abdominal simulator training as remediation, than as a requirement for all students.

Our study design also could not tell us how long the effects of the training last. We don’t have performance measures at the end of the month, so we cannot compare the effects of the simulator to the effects of the clinical experiences offered by the family medicine, internal medicine, and surgery clerkships. We did not investigate the difference between the effect of expensive, guided individual use of the simulator versus less costly group instruction, or individual sessions using the simulator guided only by its computer program. We did not quantify the cost of the simulator per student; as this study focused on individual measurement, it used researchers not teachers, and did not compare individual instruction with team or group simulator exercises.

The AbSim simulator was used both to provide the training and to assess student performance on the trained skill. To allay concern about simply “training for the test,” it would be better to have an independent assessment of change in competence. Similarly, to assess the accuracy of the simulator’s assessments, it could be used before and after a different form of training, and/or compared with an alternative valid measurement of competence.

## Conclusion

The study used a novel abdominal simulator to both assess individual students’ palpatory examination of the abdomen, and to train them using immediate and summative feedback to improve their performance. On multiple measures, the participating students showed significant improvements. Additional studies with alternative designs and measurements could assess the robustness of these gains in skill.

## Supplementary Information

**Additional file 1.**

## Data Availability

Deidentified data are available upon request from Robert Hamm, Robert-hamm@ouhsc.edu or robertmhamm@gmail.com.

## Footnotes

1 ACDET, Inc., Fort Worth, Texas, https://www.acdet-absim.com/. Author Frank Papa is co-developer and co-owner of ACDET.

2 That layer also has “bladders” that can be inflated to represent enlarged organs or abdominal wall guarding – features not used in this study. In the empty space below this layer is a movable lump with which the computer can represent various inflamed or diseased organs – also not used.
