# Supplementary material for: Effects of using an abdominal simulator to develop palpatory competencies in 3rd year medical students
Source: BMC Med Educ. 2022 Jan 26;22:63. doi: 10.1186/s12909-022-03126-y (PMC8793257; doi:10.1186/s12909-022-03126-y)
Supplement: Supplementary file 1 — Additional file 1. [file 12909_2022_3126_MOESM1_ESM.docx]

Supplemental Materials for “Effects of Using an Abdominal Simulator to Develop Palpatory Competencies in 3^rd^ Year Medical Students” by Robert M. Hamm, David M. Kelley, Jose A Medina, Noreen S. Syed, Geraint A. Harris, and Frank J. Papa.

Contents.

Appendix 1.

a. Questionnaire at Beginning and End of 4-week Family Medicine Clerkship Rotation.

b. Additional Questions at the End of Rotation.

Appendix 2. The AbSim Abdominal Simulator.

a. Manufacturer.

b. Details of Calibration.

c. Description of Subset of Tutorial Training used in the Study.

Appendix 3. Changes over Month of the Academic Year.

a. In Participation Rate and Rate of Choosing to Study with Abdominal Simulator Tutorial.

b. In Pre- and Post-Clerkship Self-Reported Confidence in One’s Abdominal Examination.

c. In Pre-Tutorial and Post-Tutorial Performance as Measured by the Abdominal Simulator.

Appendix 4. Effects of AbSim Tutorial on Confidence (self report) and Performance (simulator measured palpation performance).

a. Relations between confidence and training with the abdominal simulator.

b. Relations between student gender and confidence.

c. Tables comparing pre-training and post-training performance on depth of palpation and breadth of coverage.

Appendix 5. Bivariate relations among Confidence, Studying with AbSim Tutorial, and Performance.

a. The relation between performance and confidence.

b. Relation between changes in performance from before to after the training, and changes in confidence from the beginning to the end of the clerkship.

Appendix 6. Multivariate analyses.

a. Predictions of pre-clerkship confidence in one’s ability to examine the abdomen.

b. Predictions of studying with the abdominal simulator.

c. Predictions of performance palpating the abdomen, before the tutorial training.

d. Predictions of performance palpating the abdomen, after the tutorial training.

Appendix 7. Structural equation models.

1. Confidence data for all students.
2. Performance data for students who studied with the AbSim simulator.

Appendix 1.a. Questionnaire at Beginning and End of 4-week Family Medicine Clerkship Rotation.

How confident do you feel about identifying abdominal disease states and their respective physical findings? (circle one)

Not confident at all Unconfident Confident Very confident

How confident do you feel in using correct technique to palpate the abdomen, in your physical exam of a patient complaining of abdominal pain?

Not confident at all Unconfident Confident Very confident

How confident are you in your ability to attribute abdominal tenderness to the right organ?

Not confident at all Unconfident Confident Very confident

How confident are you in your ability to recognize muscular guarding during the abdominal exam?

Not confident at all Unconfident Confident Very confident

How confident are you in your ability to identify abdominal organomegaly?

Not confident at all Unconfident Confident Very confident

How confident are you in your ability to identify right hepatic lobe enlargement?

Not confident at all Unconfident Confident Very confident

How confident are you in your ability to use light and deep palpation?

Not confident at all Unconfident Confident Very confident

Please rank order these methods of learning how to do an accurate abdominal exam

(rank order them all: 1 = most preferred, 9 is least preferred).

___ Reading.

___ Video tutorial with animation.

___ Lecture with powerpoint.

___ Lecture with instructor demonstration on volunteer student.

___ Practicing the physical exam on other students’ abdomens.

___ Group exercise with a computerized mannequin simulator.

___ Individual practice with a computerized mannequin simulator.

___ Interacting with and examining a standardized patient.

___ Interacting with and examining a real patient.

Appendix 1.b. Additional Questions at the End of Rotation.

How much did you learn from your experience with the abdominal simulator a couple of weeks ago? (check one)

___ Nothing new.

___ I learned a little but would need to use it some more to really benefit from it.

___ I learned a little, and it was about all I would ever learn from using such a device.

___ I learned a lot.

___ I did not work with the abdominal simulator.

Would it be useful to be taught in class using an educational abdominal simulator (computerized mannequin) for demonstrations of the abdominal exam, where you could watch others doing the exam and getting feedback, though you did not personally touch the abdominal simulator? (circle one)

Yes No

Would it be useful to study on your own (without an instructor) with an educational abdominal simulator (computerized mannequin) that provides visual feedback on your manual technique?

Yes No

If you had the opportunity to study using an abdominal simulator, would you want to use it as an independent study tool, or would you want help and guidance with it? (check one)

___ I would prefer to use it independently

___ I would prefer to have a person guide me in using it

Would you recommend integrating the abdominal simulator into the Family Medicine clerkship curriculum?

Yes No

During your family medicine clerkship, how many abdominal exams have you performed on patients? ____________

Have you completed your surgery clerkship? Yes_____ No______

Have you completed your internal medicine clerkship? Yes_____ No______

Appendix 2. The AbSim Abdominal Simulator.

2.a. Manufacturer.

The AbSim Abdominal Simulator is developed and is sold by ACDET, Inc., of Fort Worth Texas (<http://absim.businesscatalyst.com/> and https://www.acdet-absim.com/). Author Frank Papa is a developer of AbSim and an owner of ACDET. Use of the simulator was provided for the purposes of this study for no charge. Dr. Papa did not analyze the data.

2.b. Details of Calibration.

The simulator’s feedback is calibrated based on the developers’ judgment as checked, over a five year period, by more than 500 practicing physicians from around the world (encountered at national and international simulation conferences), who provided AbSim’s developers with feedback representing their estimates of what constituted palpatory efforts that were light, deep, too deep and thorough. In regards to palpatory depth, their collective feedback enabled the developers to continually recalibrate AbSim’s sensor pad so that it reflected their judgments of palpation representing light, deep and too deep. In regards to thoroughness of an abdominal examination, these physicians also indicated the areas of the abdomen which, when palpated, would represent areas considered essential for demonstrating a thorough examination of the abdomen. Collectively, these experts suggested that the areas to be examined should include those overlying the following organs: gallbladder, liver, upper gastrointestinal region, pancreas, spleen, appendix, right ovary, left ovary, normal urinary bladder, distended urinary bladder, and descending colon.

2.c. Description of Subset of Tutorial Training used in the Study.

The simulator tutorial’s sequence of exercises is designed to support the student’s development of appropriate sensory/motor skills (i.e., the tactile sensation associated with motor-driven changes in the depth of the learner’s palpatory efforts). It is predicated upon two factors: the inherent tensile qualities of the simulators anterior wall, and a tensioning device which enables faculty to make adjustments in the overall tensile/resistive properties of the wall. Sensors in the wall of the simulator’s abdomen generate real-time, monitor-displayed visual feedback in response to the depth and location of the learner’s palpatory efforts. This visual feedback appears in the form of the colored dots which serve two roles.

First, the dots change colors depending upon the amount of palpatory pressure applied to the abdomen (i.e., the learner’s depth of palpation). That is, the simulator’s dot based mirroring of the depth of the learner’s palpatory efforts (gray dots=light palpation; blue dots=deep palpation; red dots=palpation which is too deep) enables the learner to see, in real time, the depth of their palpatory efforts. Second, the dots change their location in response to changes in the location of the subject’s palpatory efforts. More specifically, the dots are superimposed upon a schematic drawing of the abdomen which also displays where organs likely to cause common abdominal disorders are located. The simulator’s dot based mirroring of the learner’s palpatory efforts thereby enable the learner to see, in real time, the part of the abdomen (and the underlying organs) over which they have and have not palpated. (see Figures 1a and 3 of main paper).

The primary purpose of this visually-based corrective feedback (the outline of the abdomen, showing current depth of palpation), correlated with palpatory perception [how it feels to be pressing at that location], is formative in nature. That is, to enable the students to calibrate, in real time, their evolving sensory/motor palpatory capabilities to be congruent with the criteria used to define palpation representing appropriate depth (light, deep and too deep) and thoroughness. Emphasis on developing sensory/motor skills representing palpation which is light, deep and too deep stems from consideration of the following. Palpation which is deemed too light (by the simulator’s sensors) could lead to false negatives. It could cause the learner to believe that there was no evidence of point tenderness when in fact, the presence of a significant pathological process could have been detected by palpating to a more appropriate depth (i.e., deep palpation). Palpation which is deemed too deep (by the simulator’s sensors) could lead to false positives. It could cause the student to believe that there was tenderness when in fact no pathological process is present.

Arguably, when learners are able to objectively demonstrate that their palpatory efforts are well calibrated to or approximate what experienced clinicians would deem to be palpation which is light, deep and too deep, it could be said that they attained a level of knowledge (level 3B in Moore’s assessment rubric (Moore, Green, & Gallis, 2009)) reflecting that they know how to palpate an abdomen. When they demonstrate that their palpatory efforts are sufficient to actually detect, via palpation, underlying pathological processes when present, along with a reduced tendency to self-generate examination artifacts (i.e., create false negative and false positive palpatory findings), it would then be possible to argue that they are demonstrating a rudimentary level of abdominal palpatory competencies (level 4 in Moore’s rubric). Auditory feedback was also used in this study. Its role was to generate vocalizations of pain [randomly selected from a library of similar recordings] when the simulator’s sensors detect palpatory efforts identified as too deep.

The simulator is shipped with physician-derived, factory calibrated settings representing criteria by which the depth of a subject’s palpatory efforts are assessed as light, deep or too deep. The developing physician’s judgments as checked by multiple practicing physicians was used to establish these depth of palpation criteria. However, the simulator was also designed to enable faculty to establish their own depth criteria. The investigators conducting this study elected to utilize the factory set depth criteria. Collectively, the simulator’s training objectives and formatively directed feedback mechanisms represent 5 of the 12 features (McGaghie, Issenberg, Petrusa, & Scalese, 2010) identified as promoting learning: “(i) feedback; (ii) deliberate practice; (iii) curriculum integration [in that the students may well need it in their current clinical placement]; (v) simulation fidelity; … (xi) instructor [researcher] training.”

Appendix 3. Changes over Month of the Academic Year.

As the months of the third year of medical school pass, students start their family medicine rotation with more and more experience, including previous rotations with specialties that also instruct in the physical examination of the abdomen – surgery, internal medicine, and to a lesser extent obstetrics & gynecology and pediatrics. This accumulating experience can be expected to influence the students’ inclination to seek to study with the abdominal simulator tutorial, and their performance palpating the abdomen, as briefly reported in the main text, as well as their confidence in their abdominal skill.

3.a. Change in participation rate and rate of choosing to study with abdominal simulator tutorial.

The overall rates are shown in this table, the proportion of the 137 students who participated.

|  | **Initial Questionnaire** | **AbSim Tutorial** | **Final Questionnaire** |
| --- | --- | --- | --- |
| Proportion of Clerkship Students (out of 137) | 86.9% | 53.3% | 77.4% |
| Proportion of Those who Did Initial Questionnaire (out of 119) |  | 61.3% | 89.1% |

The researchers were not available to provide the AbSim tutorial sessions in June, although administrative staff gave the questionnaires. The lack of opportunity to do the tutorial may have caused lower student completion of the questionnaires. Excluding that month, there was no decline in the rate of participation (r(month, % initial questionnaire) = -0.05, p = 0.44) and final questionnaire (r = -0.24, p = 0.24), but the proportion of students choosing to do the AbSim tutorial declined significantly (r = -0.63, p = 0.019). These correlations involve monthly summary data, not individual student data.


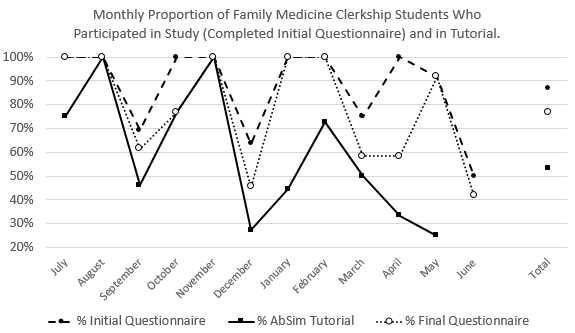


3.b. Change over the year in the students’ confidence in their technique for the physical examination of the abdomen, at the beginning and end of the family medicine clerkship.

Confidence was elicited on a 1 to 4 “self-reported competence” scale, for seven different questions (see questionnaire in Appendix 1, and detailed results below). On the first day of the clerkship, student confidence would be based on previous experience before and during medical school, including completed 3^rd^ year clerkships. At the end, all students had had 4 weeks of clinical experience and instruction in primary care practices, and some would have used the AbSim tutorial. The graph shows the confidence ratings averaged over 7 questions, and over all participating students each month. Clearly, their average confidence improved during the clerkship, as shown by the difference in the lines. Across the months of the year, the initial confidence increased significantly as expected (r(month, average confidence) = 0.701, p = 0.008; excluding June r = 0.698). However, the confidence at the end of the family medicine clerkship did not change (r = -0.06; excluding June r = 0.10; 2-tailed p = 0.345). The increase due to the month of family medicine is greater than the increase due to previous clerkship training.


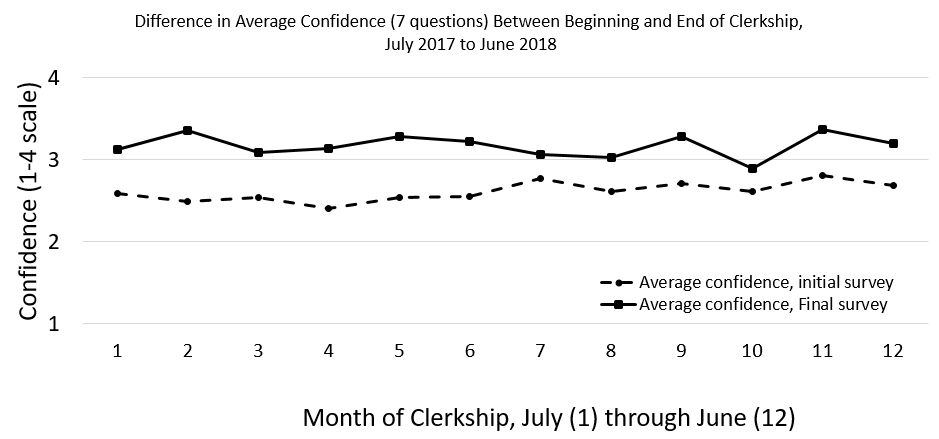


That graph, however, does not distinguish between students who did the AbSim tutorial and those who did not. We can compare temporal trends in the separate groups’ initial confidence, and their final confidence. Initial confidence would reflect the impact of their prior experiences, and might influence their choice to study with the AbSim tutorial. It can be seen in the graph below (dotted lines) that the two groups start the year with equal confidence, but then those who did not choose to use the AbSim tutorial gained confidence (r(month, initial confidence) = 0.717, p = 0.007) while those who chose to study with it did not (r = 0.133, p = 0.35). At the end of the clerkship, which for those who did not do the AbSim tutorial would reflect the influence of both the previous and the family medicine clerkships, and for those who studied with AbSim would reflect the previous and current clerkships and the tutorial, confidence in one’s abdominal exam increased nonsignificantly over the year for those who did not do the tutorial, r(month, final confidence) = 0.393, p = 0.12) and decreased nonsignificantly over the year for those who studied AbSim (r = -0.101, 2-tailed p = 0.77).


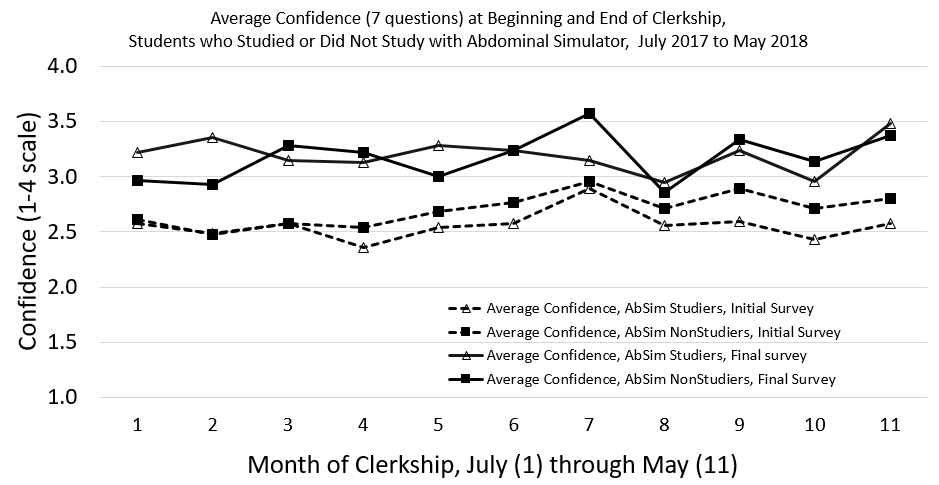


These patterns in the average monthly data suggest that the students who are less confident in their abdominal exam technique at the beginning of the family medicine clerkship were more likely to choose to study the AbSim tutorial, and that studying helped them increase their confidence to be about the same as the other students’, at the end of the month.

3.c. Change over the year in the pre-tutorial and post-tutorial measures of performance, among the students who studied with the AbSim tutorial.

Looking at the thoroughness of the student’s palpation of the abdomen, the proportion of the area that was not palpated at all decreased during the course of the academic year, both before the training (r = -0.222, p = .082, N = 62) and after (r = -.262, p= .028, N = 70). Up is worse in this graph.
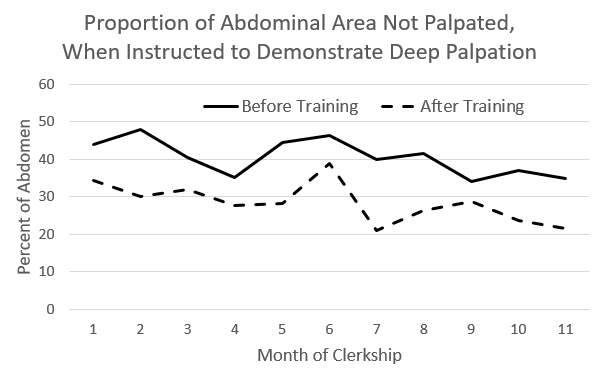


There were few temporal trends in the measures of palpation performance that focus on technique and thoroughness exploring specific organs. Of the three measures (one of calibration of the light exam, two of the deep exam) before and after training, only one (a measure of deep calibration, after training) improved significantly over the year (F = 4.35, p = .041, df = 1, N = 71), shown in the next figure (up is better) along with the same measure, before training, which did not change significantly. None of the measures of improvement due to training (i.e., calculated differences between a student’s post- and pre-training performance scores, averaged over all students each month) changed over time. Note that the amount of improvement in performance over the year is substantially less than the improvement due to the training. (Of course such improvement is measured at the most favorable time, immediately after the training). Interpretation of the data, graphed by month, must keep in mind that there were fewer students studying with AbSim in the later months.


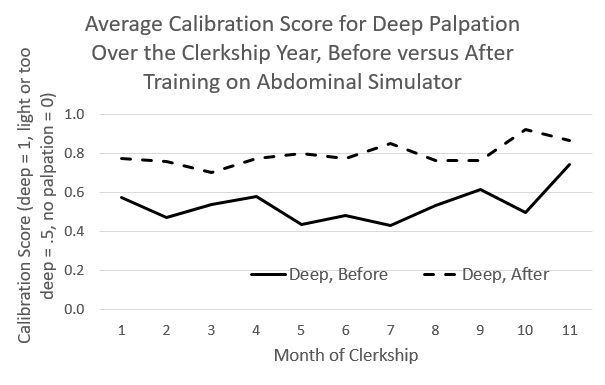


Appendix 4. Effects of AbSim Tutorial on Confidence (self-reported competence) and Competence (simulator measured palpation performance).

The validity of confidence or self-reported competence as measures of the efficacy of educational interventions has been questioned, due to low relation with more objective measures (Eva, Cunnington, Reiter, Keane, & Norman, 2004). Self-assessments may be influenced by processes of social desirability (seeking to look good or to please the teacher) or self delusion (most of us think we are better than average drivers (Weinstein, 1980)), and they vary with context and degree to which a habit of accurate self-reflection has been cultivated (Eva & Regehr, 2005). We present the effects of the family medicine clerkship month, AbSim training, and Gender on the study’s confidence measures here (see questions and response categories in Appendix 1), analyzing the individual student data rather than the monthly summaries (as done in Appendix 3).

The study posed seven questions regarding the students’ confidence in their ability to conduct the physical examination of the abdomen, each rated on a 4 point ordered category scale (Not confident at all, Unconfident, Confident, Very confident; see Appendix 1). The questions covered a variety of components and uses of the abdominal exam. Some of these were directly addressed by the AbSim training (awareness and control of depth of palpation and thoroughness of coverage, naming the organs at different locations but not providing experience of feeling an abnormality there), while others were related only indirectly. When deciding whether to work with the AbSim tutorial, students did not know which aspects would be emphasized. At the end of the month, only the students who had done the tutorial knew.

a. Relations between confidence and training with the abdominal simulator.

Confidence is both a predictor of arranging to train with the abdominal simulator, and an effect of the training. In the next figure, the difference between the bottom two curves showing the confidence at the beginning of the family medicine clerkship indicates that the students who (after making these ratings) chose to study with the AbSim had less confidence in their ability than those who opted to do without this extra, noncredit work. The difference between the top two and bottom two curves shows the impact on confidence of the family medicine month, which for the AbSim group also includes taking the tutorial. Looking at the average confidence (points on the right) as well as the individual confidence items (connected points), it reaffirms the impression that the greater increase in confidence of those who chose to take the abdominal simulator training is due to the fact that they started with less confidence.


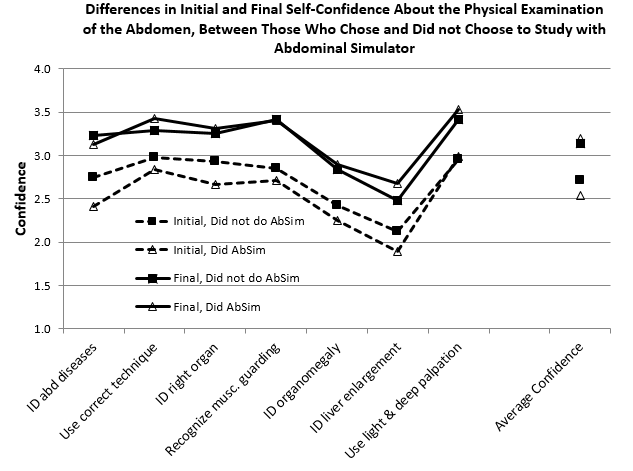


The statistical tests for various comparisons between these lines are in the 5 columns on the right (in the table on the next page). On the average (bottom row), the students who chose to study AbSim had significantly lower confidence than those who did without, and this was true for three of the specific questions (9^th^ column). After the month there were no differences in their confidence (10^th^ column). Those who studied with AbSim reported themselves to be significantly more confident at the end of their month of family medicine clerkship on every one of the 7 confidence questions, and overall (11^th^ column, all t’s > 5.7, p’s < 0.001). However, those who did not study with AbSim were also more confident at the end of the month (12^th^ column, all t’s > 2.3, p’s < 0.026). Corresponding to the lower initial confidence of the AbSim group but the lack of difference at the end of the rotation, although all the increases were statistically significant in both groups, the AbSim group’s confidence gain was significantly larger than the no-AbSim group’s (last column). This difference was statistically significant for the mean confidence (F= 8.1, p = 0.005) and for the ratings of confidence in ability to attribute tenderness to the right organ and to identify right hepatic enlargement, with the test of the difference being less than p = .10 for another three of the items. It should be noted, however, that one of the two things the AbSim training focused on was depth of palpation; and that is the question where there was the smallest difference in the improvement in confidence between the tutored and nontutored groups, as well as the smallest initial difference.

Though the effects of the family medicine clerkship were slightly higher for those who used AbSim, clearly there was also an effect of the month’s clinical experience on everyone’s confidence. Additionally, there may be a “social desirability” effect – wishing to please the professor by praising the course. The student’s names were on the surveys. Although in accord with promises made in the informed consent form, the individual responses were seen only by the administrative staff who collected the surveys and entered the data, not by the professor, the students may not have remembered or trusted this promise. The most important factor here, however, may be the fact that the students who chose to study with the abdominal simulator had lower confidence initially. As such, the AbSim group’s greater increase in confidence may be regression to the mean, rather than an effect of the AbSim tutorial. Or the tutorial may have allowed them to “catch up.” This will be explored more in Appendix 5.

|  | Initial - No AbSim.  N = 40 | | Initial - AbSim.  N = 72 | | Final - No AbSim.  N = 31 | | Final - AbSim.  N = 70 | | AbSim vs  No AbSim | | Initial vs  Final | | AbSim vs No AbSim |
| --- | --- | --- | --- | --- | --- | --- | --- | --- | --- | --- | --- | --- | --- |
| Confidence in ability… | Mn | SD | Mn | SD | Mn | SD | Mn | SD | Initial | Final | AbSim | No AbSim | Initial-Final Change |
| to identify abdominal disease states and their respective physical findings | 2.750 | 0.4935 | 2.410 | 0.4917 | 3.23 | 0.497 | 3.13 | 0.479 | F=13.0 p<0.001 | F=0.9  p=0.36 | t=10.0  p<0.001 | t=4.1  p<0.001 | F=2.8  p=0.096 |
| to use correct technique to palpate the abdomen | 2.975 | 0.3572 | 2.833 | 0.4441 | 3.29 | 0.461 | 3.43 | 0.498 | F=3.1 p=0.081 | F=1.7  p=0.19 | t=8.1  p<0.001 | t=3.4  p=0.002 | F=3.4  p=0.067 |
| to attribute abdominal tenderness to the right organ | 2.925 | 0.4743 | 2.667 | 0.5307 | 3.26 | 0.445 | 3.31 | 0.526 | F=6.8 p=0.01 | F=0.3  p=0.61 | t=8.1  p<0.001 | t=3.8  p=0.001 | F=5.1  p=0.027 |
| to recognize muscular guarding during the abdominal exam | 2.850 | 0.6222 | 2.708 | 0.5919 | 3.42 | 0.564 | 3.40 | 0.623 | F=1.0 p=0.31 | F=0.0  p=0.88 | t=8.0  p<0.001 | t=4.8  p<0.001 | F=1.3  p=0.262 |
| to identify abdominal organomegaly | 2.425 | 0.5495 | 2.250 | 0.5241 | 2.84 | 0.583 | 2.90 | 0.663 | F=2.50 p=0.12 | F=0.2  p=0.66 | t=7.2  p<0.001 | t=2.8  p=0.010 | F=3.5  p=0.065 |
| to identify right hepatic lobe enlargement | 2.125 | 0.4634 | 1.896 | 0.5499 | 2.48 | 0.724 | 2.67 | 0.696 | F=5.0 p=0.03 | F=1.5  p=0.22 | t=8.9  p<0.001 | t=2.3  p=0.026 | F=8.5  p=0.004 |
| to use light and deep palpation | 2.950 | 0.5038 | 2.986 | 0.5171 | 3.42 | 0.502 | 3.53 | 0.583 | F=0.1 p=0.7 | F=0.8  p=0.37 | t=5.7  p<0.001 | t=4.1  p<0.001 | F=0.0  p=0.946 |
| Average confidence, seven questions | 2.7143 | 0.26677 | 2.5357 | 0.27625 | 3.1336 | 0.33591 | 3.1959 | 0.38699 | F=10.8 p=0.001 | F=0.6  p=0.44 | t=12.9  p<0.001 | t=7.4  p<0.000 | F=8.1  p=0.005 |

b. Relations between student gender and confidence.

The AbSim tutorial was chosen about equally often by males (55%) and females (58%). The next figure illustrates gender differences in confidence, at the beginning and end of the month. Initially (lower two lines), the men were generally more confident than the women, with a statistically significant difference in their confidence about detecting guarding and organomegaly. Both genders reported greater confidence at the end of the month. At that point, men still were generally more confident, particularly about their technique, ability to detect organomegaly, and light and deep palpation.


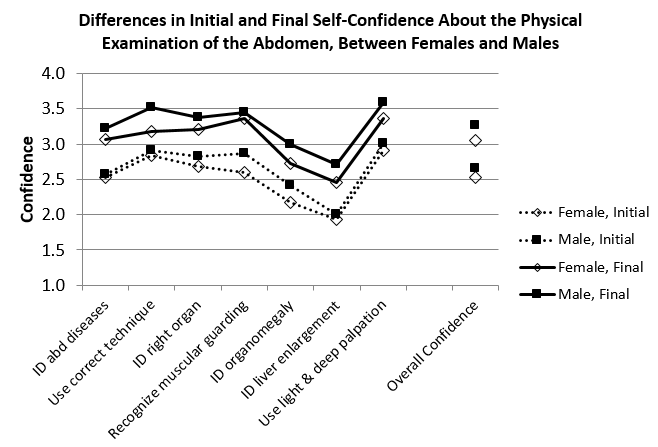


The largest effect in the graph is the difference in the students’ confidence in their ability at the beginning and end of the rotation, as noted in a previous section. Every comparison of initial and final confidence was statistically significant at p <= 0.001, for each gender. At the initial self-report, male students were statistically significantly more confident than females about their ability to recognize muscular guarding during the abdominal exam (F(1,111) = 7.85, p = 0.006), ability to identify abdominal organomegaly (F(1,111) = 5.17, p = 0.025), and the average of all 7 confidence questions (F(1,111) = 7.7, p = 0.006). At the end of the month, males were more confident than females about using correct technique to palpate the abdomen (F(1,99) = 11.6, p = 0.001) and overall (F(1,99) = 7.83, p = 0.006), and almost significantly more confident about attributing abdominal tenderness to the right organ (F(1,99) = 2.91, p = 0.091), identifying abdominal organomegaly (F(1,99) = 3.91, p = 0.051), and using light and deep palpation (F(1,99) = 3.79, p = 0.054).

c. Tables comparing pre-training and post-training performance on depth of palpation and breadth of coverage.

These tables give the numbers graphed in the table.

Depth of palpation, before and after training. (Figure 3 of main text). Percent of abdomen ("dots") palpated at each depth, when told to use light and then deep palpation. N = 61.

|  |  | Before Training | After Training |
| --- | --- | --- | --- |
| Percent of abdomen not palpated | Mean | 40.9 | 28.1 |
|  | Stand. Dev. | 12.4 | 9.6 |
| Percent of abdomen palpated only lightly | Mean | 26.7 | 38.1 |
|  | Stand. Dev. | 10.6 | 9.7 |
| Percent of abdomen palpated deeply | Mean | 25.1 | 31.5 |
|  | Stand. Dev. | 11.7 | 12.0 |
| Percent of abdomen palpated too deeply | Mean | 7.1 | 2.3 |
|  | Stand. Dev. | 7.8 | 2.7 |

Light palpation score for each organ and for regions of abdomen, before and after training. (Figure 5-a of paper).

|  | Overall |  | Upper Abdomen | Lower Abdomen | | Right Upper Quadrant | Upper Midline | Left Upper Quadrant | | | Right Lower Quadrant | Lower Midline | Left Lower Quadrant |
| --- | --- | --- | --- | --- | --- | --- | --- | --- | --- | --- | --- | --- | --- |
| Before, Light | 0.46 |  | 0.31 | 0.59 |  | 0.41 | 0.11 | 0.49 |  | 0.67 | | 0.48 | 0.61 |
| Before, Deep | 0.43 |  | 0.32 | 0.53 |  | 0.47 | 0.11 | 0.45 |  | 0.53 | | 0.50 | 0.55 |
| Baseline for Training | 0.49 |  | 0.39 | 0.57 |  | 0.51 | 0.20 | 0.55 |  | 0.59 | | 0.50 | 0.61 |
| Post Training Comparison | 0.59 |  | 0.52 | 0.65 |  | 0.62 | 0.37 | 0.61 |  | 0.66 | | 0.64 | 0.66 |
| After, Light | 0.78 |  | 0.67 | 0.88 |  | 0.84 | 0.46 | 0.76 |  | 0.86 | | 0.89 | 0.88 |
| After, Deep | 0.56 |  | 0.51 | 0.60 |  | 0.56 | 0.40 | 0.64 |  | 0.61 | | 0.60 | 0.61 |

Deep palpation score for each organ and for regions of abdomen, before and after training (Figure 5-b of paper).

|  | Overall |  | Upper Abdomen | Lower Abdomen |  | Right Upper Quadrant | Upper Midline | Left Upper Quadrant | | Right Lower Quadrant | Lower Midline | Left Lower Quadrant |
| --- | --- | --- | --- | --- | --- | --- | --- | --- | --- | --- | --- | --- |
| Before, Deep | 0.53 |  | 0.36 | 0.67 |  | 0.52 | 0.12 | 0.55 |  | 0.74 | 0.57 | 0.69 |
| Baseline for Training | 0.57 |  | 0.42 | 0.70 |  | 0.57 | 0.16 | 0.66 |  | 0.78 | 0.58 | 0.73 |
| Post Training Comparison | 0.74 |  | 0.66 | 0.81 |  | 0.81 | 0.42 | 0.85 |  | 0.81 | 0.82 | 0.79 |
| After, Deep | 0.78 |  | 0.71 | 0.85 |  | 0.87 | 0.48 | 0.83 |  | 0.87 | 0.83 | 0.84 |

Comparison of depth scores by males and females, before and after training. (Figure 6 of paper).

|  |  | Before | |  | After | |
| --- | --- | --- | --- | --- | --- | --- |
|  |  | Male (N= 41) | Female (N = 31) |  | Male (N= 41) | Female (N = 31) |
| Light Ideal | Mean | 0.50 | 0.41 |  | 0.78 | 0.79 |
|  | SD | 0.21 | 0.19 |  | 0.12 | 0.10 |
| Deep Ideal, Baseline vs Comparison | Mean | 0.58 | 0.46 |  | 0.62 | 0.51 |
|  | SD | 0.19 | 0.14 |  | 0.18 | 0.14 |
| Deep Ideal | Mean | 0.79 | 0.14 |  | 0.81 | 0.75 |
|  | SD | 0.10 | 0.17 |  | 0.09 | 0.13 |

Appendix 5. Relations among Confidence, Studying with AbSim Tutorial, and Performance.

We have seen that the students who took the abdominal simulator tutorial had greater increases in confidence than those who did not, but this is confounded with the facts that they started with lower confidence (it could be regression to the mean), that those with lower confidence were more likely to use the AbSim tutorial, and that fewer students chose the tutorial as the year went by. How accurate is that confidence – does it really reflect improved performance? And are there different analyses that might clarify whether studying with the simulator really increases confidence in one’s ability? We will look first at the correlations of confidence with the objective scores as measured by AbSim, which is only available for those who used it. Then in Appendix 6 we will see what can be learned from more complex statistical models. We will use the individual data to see what prior characteristics and experiences predict each subsequent behavioral or attitudinal measurement, looking at both the direct relations between pairs of variables, and multivariate predictive models. Then in Appendix 7 we will explore what can be learned from an integrative, structural equation model.

a. The relation between performance and confidence.

Is the students’ confidence in their abdominal examination (specifically, in their palpation technique) related to their measured performance (specifically, palpatory calibration)? This is addressed with the correlation between confidence and performance, before and after the training session, and the correlation between the amounts of change in self confidence from beginning to end of the clerkship, and change in performance from before to after the training. This table shows the relation between

| In each cell:  Correlation  p-value | Light Ideal Score Given Light Instructions, Before Training.  N = 72 | Deep Ideal Score Given Deep Instructions, Before Training.  N = 72 | Deep Ideal Score Given Both Light and Deep Instructions (Baseline), Before Training. N = 60 | Average of Light and Deep Calibration Scores When So Instructed, Before. N = 72 |
| --- | --- | --- | --- | --- |
| Initial Confidence about identifying abdominal disease states and their respective physical findings | 0.14 | 0.15 | 0.12 | 0.16 |
|  | 0.23 | 0.21 | 0.38 | 0.17 |
| Initial Confidence about using correct technique to palpate the abdomen | 0.11 | 0.19 | 0.13 | 0.17 |
|  | 0.35 | 0.10 | 0.32 | 0.16 |
| Initial Confidence about your ability to attribute abdominal tenderness to the right organ | -0.06 | -0.06 | -0.08 | -0.07 |
|  | 0.59 | 0.60 | 0.55 | 0.55 |
| Initial Confidence about ability to recognize muscular guarding during the abdominal exam | -0.07 | -0.10 | **-0.25** | -0.10 |
|  | 0.56 | 0.38 | **0.05** | 0.42 |
| Initial Confidence about your ability to identify abdominal organomegaly | 0.08 | 0.19 | 0.03 | 0.15 |
|  | 0.50 | 0.10 | 0.83 | 0.21 |
| Initial Confidence about your ability to identify right hepatic lobe enlargement | 0.08 | 0.06 | 0.01 | 0.08 |
|  | 0.50 | 0.61 | 0.95 | 0.50 |
| Initial Confidence about your ability to use light and deep palpation | 0.01 | 0.07 | 0.06 | 0.04 |
|  | 0.94 | 0.57 | 0.64 | 0.73 |
| Average confidence, seven questions, initial survey | 0.07 | 0.12 | -0.02 | 0.11 |
|  | 0.56 | 0.31 | 0.90 | 0.38 |

the students’ self-reported confidence in various aspects of their ability to perform an abdominal examination (plus an overall score) and four measures of their performance, as measured by the abdominal simulator before they received any training. Only one of 32 correlations is statistically significant, suggesting that there is essentially no relation between objective performance and student self-assessment of ability.

b. Relation between changes in performance from before to after the training, and changes in confidence from the beginning to the end of the clerkship.

It might be argued that though students have idiosyncratic interpretations of the scales they are asked to use to assess their ability, or to express confidence in it, they may be able to use the scale consistently to assess their competence. Thus, although at one moment ratings of students’ confidence in their ability may not be correlated with performance, changes in performance (due to training, for example) may be reflected in changes in the students’ self assessments of competence. The next table presents correlations between changes in self-confidence about various aspects of ability to conduct the physical exam of the abdomen (between the beginning and the end of the family medicine clerkship month) and changes in performance as measured by the simulator (from immediately before to immediately after the training). Only one of 56 correlations is statistically significant, involving variables not plausibly related to the training (change in calibration of one’s palpation when instructed to palpate deeply, and change in confidence about ability to identify abdominal organomegaly; irrelevant because the simulator did not provide any experience feeling abnormal organs). Another relation, between the reduction in the area palpated too deeply and confidence in distinguishing light and deep palpation, was explicitly trained so there should have been a relation, but it was only marginally significant (p = 0.07). This confirms the impression that there is no relation between one’s judgment of one’s ability (measured by self-confidence) and one’s actual ability. Still one could argue this is not a good test of that relationship, which is much relied on in medical education research, because the objective measures were related to only a few of the self-confidence questions, and the second confidence measure was taken three weeks after the training and could have been affected by intervening clinical experiences and training.

| In each cell:  Correlation  p-value | Change in 'light ideal' score, all organs, Pre to Post training, Light Instructions | Change in 'deep ideal' score, all organs, Pre to Post training, Deep Instructions | Change in Deep Ideal score, all organs, Baseline to Comparison | Average, all CalibrationImprove-ment scores | Change in proportion of abdomen not palpated, between baseline and comparison, after both a light and a deep pass (should decrease) | Change in proportion of abdomen palpated too deeply, between baseline and comparison, after both a light and a deep pass (should decrease) | Change in proportion of abdomen deeply palpated, between baseline and comparison, after both a light and a deep pass (should increase) |
| --- | --- | --- | --- | --- | --- | --- | --- |
| Number of students | 70 | 70 | 59 | 70 | 60 | 60 | 60 |
| Change in Confidence about identifying abdominal disease states and their respective physical findings | 0.09 | 0.15 | 0.02 | 0.14 | -0.04 | 0.02 | -0.10 |
|  | 0.44 | 0.22 | 0.88 | 0.25 | 0.79 | 0.85 | 0.43 |
| Change in Confidence about using correct technique to palpate the abdomen | 0.05 | 0.12 | 0.01 | 0.10 | -0.11 | 0.13 | 0.06 |
|  | 0.65 | 0.34 | 0.93 | 0.43 | 0.42 | 0.32 | 0.63 |
| Change in Confidence about your ability to attribute abdominal tenderness to the right organ | -0.13 | -0.02 | -0.10 | -0.09 | 0.06 | -0.05 | -0.14 |
|  | 0.30 | 0.89 | 0.44 | 0.45 | 0.67 | 0.71 | 0.29 |
| Change in Confidence about your ability to recognize muscular guarding during the abdominal exam | 0.08 | 0.00 | -0.06 | 0.05 | -0.12 | 0.12 | -0.01 |
|  | 0.52 | 0.99 | 0.64 | 0.68 | 0.36 | 0.36 | 0.96 |
| Change in Confidence about your ability to identify abdominal organomegaly | 0.03 | **0.33** | 0.08 | 0.19 | -0.08 | 0.09 | -0.04 |
|  | 0.83 | **0.01** | 0.53 | 0.12 | 0.53 | 0.50 | 0.74 |
| Change in Confidence about your ability to identify right hepatic lobe enlargement | -0.03 | 0.08 | 0.06 | 0.02 | 0.04 | 0.12 | 0.02 |
|  | 0.81 | 0.53 | 0.66 | 0.86 | 0.78 | 0.35 | 0.87 |
| Change in Confidence about your ability to use light and deep palpation | 0.11 | 0.14 | 0.11 | 0.15 | -0.04 | **0.24** | 0.16 |
|  | 0.36 | 0.26 | 0.41 | 0.23 | 0.73 | **0.07** | 0.22 |
| Change in Average Confidence, all seven questions | 0.05 | 0.18 | 0.03 | 0.13 | -0.07 | 0.16 | 0.00 |
|  | 0.69 | 0.13 | 0.82 | 0.29 | 0.60 | 0.21 | 0.97 |

Appendix 6. Multivariate analyses.

We have repeated measures on multiple confidence questions and multiple performance measures, for different numbers of students, along with information about their previous clerkships and their gender. The graph indicates the relations that we will analyze with multiple linear regression or multiple logistic regression. The arrows indicate the variables that predict each variable. Predictions of pre-clerkship and post-clerkship confidence and of studying with the abdominal simulator can be modeled with all students who participated by filling out the questionnaires. Predictions involving any measures of confidence, however, can only be modeled with those students who studied with the AbSim tutorial.

Gender

Pre-FM Clerkship Experience (Int Med, Surg, and months of 3^rd^ year)

Pre-Tutorial Competence

Pre-Clerkship Confidence

Abdominal Simulator Tutorial

Post-Tutorial Competence

Post-Clerkship Confidence

a. Predictions of pre-clerkship confidence in one’s ability to examine the abdomen.

Multiple linear regression using gender, previous surgical clerkship, previous internal medicine clerkship, and month of the family medicine rotation to predict the 7 individual confidence questions, and their average, at the beginning of the clerkship, showed that in the context of the other predictors internal medicine and rotation were never statistically significant. The number of students having all required data in these regressions is 94. The unstandardized regression weights are given in the next table, with the statistically significant predictors highlighted. Confidence was a 4 level ordered categorical variable, the clerkships were measured with 1 if it had been done previously or 0, and the gender was measured with male = 1 and female = 2, so that a negative regression coefficient indicated males had higher confidence.

| Initial Confidence about: | B for Surgery clerkship | B for Gender |
| --- | --- | --- |
| identifying abdominal disease states and their respective physical findings | 0.49 | -0.06 |
| using correct technique to palpate the abdomen | 0.02 | -0.10 |
| your ability to attribute abdominal tenderness to the right organ | **0.40** | **-0.24** |
| your ability to recognize muscular guarding during the abdominal exam | **0.31** | **-0.34** |
| your ability to identify abdominal organomegaly | 0.11 | **-0.24** |
| your ability to identify right hepatic lobe enlargement | 0.21 | -0.12 |
| your ability to use light and deep palpation | 0.08 | -0.12 |
| Average initial confidence | **0.23** | **-0.18** |

b. Predictions of studying with the abdominal simulator.

Low confidence predicts choosing to get training. In bivariate comparisons, predictors of choosing to do the abdominal simulator training (Q1) included the month of the third year (as noted above), several of the individual self-confidence questions, whether the student had previously completed an internal medicine clerkship, and just one of the preference rankings for alternative study methods (dislike of reading).^[[1]](#footnote-1)^ However, all the confidence ratings were correlated with each other, and later in the year it was of course more likely the student already had had internal medicine. In a stepwise logistic regression considering all variables at once, only the month (B = -0.165, OR = 0.848 for each subsequent month, p = 0.022) and one or two of the confidence questions (initial confidence about identifying abdominal disease states and their respective physical findings, B = -1.155, OR = 0.315 for each unit on the 1 to 4 confidence scale, p = 0.012; initial confidence about using correct technique to palpate the abdomen, B = -1.183, OR = 0.307, p = 0.051) contributed to predicting whether students worked with the simulator. The table below shows the results when all variables were entered. On the left, confidence was represented by a single variable, its average, and the rotation and the average confidence each were statistically significant. The later in the year, and the more confident the student at the outset of the clerkship, the less likely the student would study with the abdominal simulator. On the right, confidence was represented by each of the 7 individual questions. Here the rotation month, whether surgery had already been experienced, and a general question about one’s confidence “identifying abdominal disease states and their respective physical findings” each predicted not taking the tutorial.

|  | B | Sig. | Exp(B) | B | Sig. | Exp(B) |
| --- | --- | --- | --- | --- | --- | --- |
| Gender | 0.427 | 0.460 | 1.533 | 0.466 | 0.456 | 1.594 |
| Rotation Month | **-0.209** | **0.039** | **0.812** | **-0.216** | **0.044** | **0.806** |
| Internal Medicine | 0.298 | 0.643 | 1.347 | 0.113 | 0.869 | 1.119 |
| Surgery | -0.883 | 0.170 | 0.413 | **-1.414** | **0.063** | **0.243** |
| Average Confidence | **-2.319** | **0.021** | **0.098** |  |  |  |
| Confidence about… |  |  |  |  |  |  |
| identifying abdominal disease states and their respective physical findings |  |  |  | **-1.360** | **0.038** | **0.257** |
| using correct technique to palpate the abdomen |  |  |  | -0.877 | 0.237 | 0.416 |
| your ability to attribute abdominal tenderness to the right organ |  |  |  | -0.728 | 0.233 | 0.483 |
| your ability to recognize muscular guarding during the abdominal exam |  |  |  | -0.084 | 0.883 | 0.920 |
| your ability to identify abdominal organomegaly |  |  |  | -0.285 | 0.583 | 0.752 |
| your ability to identify right hepatic lobe enlargement |  |  |  | -0.292 | 0.583 | 0.747 |
| your ability to use light and deep palpation |  |  |  | 0.634 | 0.237 | 1.886 |

c. Predictions of performance palpating the abdomen, before the tutorial training.

This includes only the students who did the tutorial. Three measures of performance, from three separate palpations the student did of the entire abdomen, are analyzed in parallel. The first is the measure of light palpation, when they were instructed to use light palpation. The second is the measure of deep palpation, following deep instructions. The third was the baseline record taken during the tutorial sequence, when the students were asked to make two passes over the abdomen, first demonstrating their light technique, and then their deep technique. Females had lower performance on each measure. Those who had done the internal medicine clerkship had lower measures on the baseline measure. The only confidence measure that statistically significantly predicted performance, in the

context of the other measures, was the self-reported confidence in their ability to recognize muscular guarding, negatively related to their performance on the deep ideal. The item asking about confidence identifying abdominal disease states and their physical findings was significant at p < 0.10 on both the deep measures. When the average performance was used as a predictor, instead of the 7 individual confidence items, it did not statistically significantly predict any of the three performance measures. When the average of the three performance measures was predicted, the results were very similar to those of the deep ideal, deep instructions model.

|  | Light Ideal, Light Instructions | | |  | Deep Ideal, Deep Instructions | | |  | Baseline, Light then Deep Instructions | | |
| --- | --- | --- | --- | --- | --- | --- | --- | --- | --- | --- | --- |
|  | B | t | Sig. |  | B | t | Sig. |  | B | t | Sig. |
| Gender | **-0.121** | **-2.250** | **0.028** |  | **-0.140** | **-3.308** | **0.002** |  | **-0.177** | **-4.362** | **0.000** |
| Int Med | 0.067 | 0.975 | 0.333 |  | -0.028 | -0.517 | 0.607 |  | **-0.128** | **-3.704** | **0.000** |
| Surgery | 0.020 | 0.252 | 0.802 |  | 0.002 | 0.039 | 0.969 |  | 0.064 | 1.349 | 0.183 |
| Rotation | 0.008 | 0.746 | 0.459 |  | 0.009 | 1.073 | 0.288 |  | -0.031 | -0.595 | 0.554 |
| Confidence about… |  |  |  |  |  |  |  |  |  |  |  |
| identifying abdominal disease states and their respective physical findings | 0.081 | 1.276 | 0.207 |  | 0.095 | 1.911 | 0.061 |  | 0.102 | 1.736 | 0.088 |
| using correct technique to palpate the abdomen | 0.080 | 1.224 | 0.226 |  | 0.067 | 1.306 | 0.197 |  | 0.001 | 0.080 | 0.936 |
| your ability to attribute abdominal tenderness to the right organ | -0.058 | -1.176 | 0.244 |  | -0.049 | -1.256 | 0.214 |  | 0.028 | 0.558 | 0.579 |
| your ability to recognize muscular guarding during the abdominal exam | -0.073 | -1.588 | 0.118 |  | **-0.089** | **-2.446** | **0.017** |  | -0.055 | -1.473 | 0.146 |
| your ability to identify abdominal organomegaly | -0.040 | -0.687 | 0.495 |  | 0.025 | 0.556 | 0.580 |  | -0.013 | -0.300 | 0.765 |
| your ability to identify right hepatic lobe enlargement | 0.056 | 1.082 | 0.284 |  | 0.014 | 0.347 | 0.730 |  | 0.011 | 0.292 | 0.771 |
| your ability to use light and deep palpation | -0.024 | -0.438 | 0.663 |  | 0.001 | 0.016 | 0.988 |  | 0.021 | 0.502 | 0.618 |

Exactly the same procedures were repeated after the tutorial, though in a different order, with the comparison assessment (deep ideal measure, following instructions to do a light and a deep palpatory exam), then light, then deep. Interestingly, the correlations among these measures were less after the tutorial than before.

Correlations among palpatory performance measures prior to training.

| N = 72 | Deep Ideal, Deep Instructions | Baseline | Average, Before |
| --- | --- | --- | --- |
| Light Ideal, Light Instructions | **0.587** | **0.580** | **0.909** |
| Deep Ideal, Deep Instructions |  | **0.791** | **0.871** |
| Baseline |  |  | **0.754** |

Correlations among palpatory performance measures after training. Those who scored best when asked to show their light palpation were no longer the same people who scored best when asked to do their deep palpation.

| N = 72 | Deep Ideal, Deep Instructions | Comparison | Average, After |
| --- | --- | --- | --- |
| Light Ideal, Light Instructions | 0.136 | 0.053 | **0.738** |
| Deep Ideal, Deep Instructions |  | **0.434** | **0.768** |
| Comparison |  |  | **0.329** |

Correlations between the pre-tutorial and post-tutorial measures. The only measure with statistically significant stability is the baseline – comparison, with a similar correlation between pre-baseline and post deep ideal measures. The light ideal, deep ideal, and average were not significantly stable.

| N = 72 | Light Ideal, Light Instructions, After | Deep Ideal, Deep Instructions, After | Comparison, After | Average, After |
| --- | --- | --- | --- | --- |
| Light Ideal, Light Instructions, Before | -0.011 | 0.192 | 0.170 | 0.124 |
| Deep Ideal, Deep Instructions, Before | -0.051 | 0.201 | 0.139 | 0.104 |
| Baseline, Before | -0.050 | **0.265** | **0.248** | 0.148 |
| Average, Before | -0.033 | 0.220 | 0.174 | 0.129 |

d. Predictions of performance palpating the abdomen, after the tutorial training.

After the tutorial, the gender difference was no longer significant on the light ideal and deep ideal measures, though it persisted with the Comparison. As noted with the correlations, the pre-tutorial performance did not predict the post-tutorial performance. Additionally, there was no influence of the pre-family medicine clerkships nor of the students’ overall pre-clerkship confidence. When the 7 individual confidence questions were used, rather than their average, the only significant predictor was the student’s initial confidence about their ability to use light and deep palpation, B= 0.067, t = 2.286, p = 0.025. The higher their initial confidence in their ability to use light and deep palpation, the better their performance on one of the three measures of light or deep palpation. We also calculated the change score, and predicted it from the above variables and the pre score (not shown). Consistent with other findings, only the pre-tutorial performance predicted the change in performance – lower initial performance predicted a greater increase. Analysis cannot tell us if this is due to regression to the mean, or to greater learning, or a ceiling effect.

|  | Light Ideal, Light Instructions, After | | |  | Deep Ideal, Deep Instructions, After | | |  | | Comparison, Light then Deep Instructions, After | | | |
| --- | --- | --- | --- | --- | --- | --- | --- | --- | --- | --- | --- | --- | --- |
|  | B | t | Sig. |  | B | t | Sig. |  | B | | t | Sig. |  |
| Gender | 0.004 | 0.128 | 0.899 |  | -0.040 | -1.367 | 0.176 |  | **-0.080** | | **-2.248** | **0.028** |  |
| Internal Medicine | -0.009 | -0.236 | 0.814 |  | 0.011 | 0.288 | 0.775 |  | 0.036 | | 0.841 | 0.403 |  |
| Surgery | 0.012 | 0.308 | 0.759 |  | 0.017 | 0.445 | 0.658 |  | 0.033 | | 0.719 | 0.475 |  |
| Rotation | 0.005 | 0.870 | 0.388 |  | 0.005 | 0.991 | 0.325 |  | -0.005 | | -0.841 | 0.404 |  |
| Average Confidence, Pre-Clerkship | 0.007 | 0.136 | 0.892 |  | -0.009 | -0.175 | 0.861 |  | 0.074 | | 1.163 | 0.249 |  |
| Light Ideal, Before | -0.021 | -0.309 | 0.758 |  |  |  |  |  |  | |  |  |  |
| Deep Ideal, Before |  |  |  |  | 0.097 | 1.166 | 0.248 |  |  | |  |  |  |
| Baseline, Light & Deep |  |  |  |  |  |  |  |  | 0.143 | | 1.442 | 0.154 |  |

Predictions of post-clerkship confidence, all students (N = 97), are analyzed in the table below. To predict each confidence measure (column label), the pre-clerkship judgment of the same question was used, along with the experience variables. Statistically significant (p < 0.05) B’s are in bold. If 0.10 > p > 0.05, it is in bold italic. Doing the palpation training on AbSim did not affect end of clerkship confidence. Gender was related to one item significantly, and to the average, and to three items with marginal significance. Females were less confident at the end of the month. Previously doing an internal medicine or a surgery rotation was related to one of the seven items. For three of the items, and the average

|  | Identifying abdominal disease | using correct technique | attributing abdominal tenderness | recognizing muscular guarding | identifying abdominal organomegaly | identifying right hepatic lobe enlargement | using light and deep palpation | Average confidence, preclerkship |
| --- | --- | --- | --- | --- | --- | --- | --- | --- |
| Doing AbSim tutorial | -0.057 | 0.156 | 0.084 | 0.010 | 0.143 | 0.252 | 0.116 | 0.104 |
| Gender | ***-0.184*** | **-0.360** | -0.179 | -0.098 | -0.226 | ***-0.251*** | ***-0.214*** | **-0.207** |
| Intern Med | -0.011 | **0.273** | 0.062 | 0.093 | -0.132 | -0.077 | 0.036 | -0.007 |
| Surgery | **0.285** | -0.041 | 0.135 | ***0.274*** | 0.066 | 0.164 | 0.064 | ***0.165*** |
| Rotation | -0.015 | -0.015 | -0.025 | ***-0.045*** | 0.004 | -0.036 | 0.000 | -0.020 |
| Initial confidence about: |  |  |  |  |  |  |  |  |
| identifying abdominal disease states and their respective physical findings | 0.139 |  |  |  |  |  |  |  |
| using correct technique to palpate the abdomen |  | 0.168 |  |  |  |  |  |  |
| your ability to attribute abdominal tenderness to the right organ |  |  | ***0.199*** |  |  |  |  |  |
| your ability to recognize muscular guarding during the abdominal exam |  |  |  | **0.343** |  |  |  |  |
| your ability to identify abdominal organomegaly |  |  |  |  | **0.295** |  |  |  |
| your ability to identify right hepatic lobe enlargement |  |  |  |  |  | **0.400** |  |  |
| your ability to use light and deep palpation |  |  |  |  |  |  | 0.037 |  |
| Average confidence, pre-clerkship |  |  |  |  |  |  |  | **0.299** |

confidence, the pre-clerkship confidence was a significant predictor of the post-clerkship confidence. It should be acknowledged that due to intercorrelations, which experience factor (internal medicine, surgery, rotation) is significant varies according to what else is in the model.

We also explored whether the interaction of initial confidence with whether one did the AbSim is statistically significant, as that is key to our interpretation that the end confidence is no different between AbSim studiers and non-studiers because the non-studiers started out with lower confidence, and that for them studying with AbSim increased their confidence. This table shows that that particular interaction is not statistically significant. We also included the interaction of gender and initial confidence, and the three-way interaction. Note that this report is only for the “total” confidence, not for any particular item, but we already know that they are highly correlated: item distinctions were not particularly attended by the students.

| **Tests of Between-Subjects Effects** | | | | | |
| --- | --- | --- | --- | --- | --- |
| Dependent Variable: EndConfTotal Average confidence, seven questions, Final survey | | | | | |
| Source | Type III Sum of Squares | df | Mean Square | F | Sig. |
| Corrected Model | 2.814^a^ | 7 | .402 | **3.312** | **.004** |
| Intercept | 3.972 | 1 | 3.972 | **32.731** | **.000** |
| DidAbSim | .327 | 1 | .327 | 2.692 | .104 |
| nGender | .822 | 1 | .822 | **6.774** | **.011** |
| InitConfTotal | .740 | 1 | .740 | **6.101** | **.015** |
| nSurgClerk | .438 | 2 | .219 | 1.803 | .171 |
| nRotation | .383 | 1 | .383 | 3.159 | .079 |
| DidAbSim * InitConfTotal | .277 | 1 | .277 | 2.279 | .135 |
| Error | 10.923 | 90 | .121 |  |  |
| Total | 1005.224 | 98 |  |  |  |
| Corrected Total | 13.737 | 97 |  |  |  |
| a. R Squared = .205 (Adjusted R Squared = .143) | | | | | |

The next table shows predictions of the same post-clerkship confidence ratings, from the same variables (except whether did AbSim – all did) plus the pre-tutorial and post-tutorial measures of the students’ palpation performance. These analyses were only done with those who did the AbSim tutorial. The performance palpating with light instructions before the tutorial was negatively correlated with one of the end of clerkship confidence questions (using light and deep palpation), and light performance after the tutorial was negatively correlated with the average and with one of the questions, and marginally with two additional questions. There is a significant interaction of the light ideal, after, with gender, in

the prediction of the average confidence. Among male students, the poorer their performance the higher their confidence at the end of the clerkship (r = 0.38, p = 0.015). The corresponding correlation among the females was nonsignificantly positive (r = 0.05). Performance with deep palpation instructions after training was marginally, positively correlated with one question (identifying organomegaly). In sum, the relation between performance and confidence is weak and shows up in unpredictable ways on just a few of the items.

Prediction of post-clerkship confidence.

|  | Identifying abdominal disease states | using correct technique to palpate the abdomen | attributing abdominal tenderness | recognizing muscular guarding | identifying abdominal organomegaly | identifying right hepatic lobe enlargement | using light and deep palpation | Average confidence, pre-clerkship |
| --- | --- | --- | --- | --- | --- | --- | --- | --- |
| Gender | ***-0.266*** | **-0.354** | -0.249 | -0.210 | -0.245 | -0.284 | -0.167 | **-0.265** |
| Intern Med | -0.083 | -0.088 | -0.088 | -0.091 | -0.156 | 0.021 | 0.045 | -0.054 |
| Surgery | 0.268 | ***0.295*** | 0.306 | **0.665** | -0.071 | 0.156 | -0.007 | ***0.251*** |
| Rotation | -0.008 | -0.023 | -0.036 | **-0.073** | 0.006 | -0.044 | 0.020 | -0.026 |
| Light Ideal, Before | -0.024 | -0.210 | 0.106 | -0.490 | -0.023 | -0.026 | **-1.125** | -0.244 |
| Deep Ideal, Before | -0.467 | 0.345 | 0.052 | 1.052 | -0.905 | -0.248 | -0.614 | -0.059 |
| Deep Ideal, Baseline | 0.007 | -0.135 | -0.718 | -1.279 | -0.518 | -0.296 | 0.859 | -0.355 |
| Light Ideal, After | -0.641 | -0.015 | ***-1.048*** | -0.268 | **-1.912** | ***-1.554*** | -0.684 | **-0.884** |
| Deep Ideal, After | -0.106 | 0.426 | 0.712 | 0.429 | ***1.568*** | 0.548 | 0.171 | 0.522 |
| Deep Ideal, Comparison | -0.434 | -0.349 | -0.568 | -0.501 | -0.345 | 0.105 | 0.728 | -0.218 |
| Initial confidence, same question | 0.199 | 0.111 | 0.142 | 0.155 | 0.389 | **0.372** | -0.036 | 0.159 |

To see if studying with the abdominal simulator increased student confidence and preference for such a study method, compared to students who did not arrange a simulator session (Q2), change scores were compared. In analyses of change in each confidence question, controlling for gender and rotation month, those who had a simulator session had larger increases in confidence (p < .05) for two of the 7 confidence statements, “Using correct technique to palpate the abdomen” and “Ability to identify right hepatic enlargement”, as well as in the average of all 7 confidence ratings. However, inspection of the graph (Figure 2) shows that both groups had similar confidence at the end of the

month, and suggests again that those who used the abdominal simulator may have had greater confidence increases because their initial confidence was lower. Analyses predicting the confidence at the end of the month from the initial confidence and whether the student worked with the abdominal simulator showed that the initial confidence but not studying with the simulator predicted the final confidence. We entertain the hypothesis that this may be due to the fact that the those with low confidence may choose to do AbSim, and when they learn more it brings them up to the level (of confidence) that the others already have. As an attempt to test this, we included an interaction term between initial confidence and doing AbSim, in the prediction of final confidence – did the initial confidence of those who took the AbSim tutorial have a different relation with the final confidence, than among those who did not do the tutorial? This term was not statistically significant in the analysis for predicting any of the final confidence items. However, it does not really test our hypothesis.

Appendix 7. Structural equation models.

The analyses presented in Appendix 6 looked at the multiple predictors of each box that arrows point to, in the following graph. We identified some weak relations between performance and confidence for those who studied with the abdominal simulator, and yet a lack of difference in final confidence between those who studied and did not study with the simulator. Structural equation modeling, which provides a method of modelling all relations simultaneously, may provide additional insights. Because the performance measures exist only for those who took the tutorial, it is not possible to include both the effects of doing versus not doing AbSim and the performance measures in the same model. Hence we model two different sets of variables with two different, overlapping sets of students – all students (addressing what affects confidence and participation), and those who studied the simulator’s tutorial (addressing what affects performance as measured by the AbSim, and improvement, including for this subset the effect of performance on confidence).

Gender

Pre-FM Clerkship Experience (Int Med, Surg, and months of 3^rd^ year)

Pre-Tutorial Performance

Pre-Clerkship Confidence

Abdominal Simulator Tutorial

Post-Tutorial Performance

Post-Clerkship Confidence

Appendix 7a. The first structural equation model excludes the performance measures, and includes all participating students (all who filled out the initial and final questionnaires). With this analysis, we can address the speculation that the initially less confident students chose to study with the abdominal simulator, while those who used the simulator would show more confidence at the end of the clerkship.

Gender

Pre-FM Clerkship Experience (Int Med, Surg, and months of 3^rd^ year)

Pre-Clerkship Confidence

Abdominal Simulator Tutorial

Post-Clerkship Confidence

Below is its representation in the AMOS SEM program. This graph shows the influence of doing the Abdominal Simulator Tutorial on every question about the student’s confidence. The model fits marginally well (RMSEA < 0.06; p = 0.05; where good fits are defined as RMSEA < 0.05 and p > 0.05), because it includes every reasonable relation. On the left are each of the 7 confidence measures, at the beginning of the month. On the right are the same variables at the end of the clerkship.


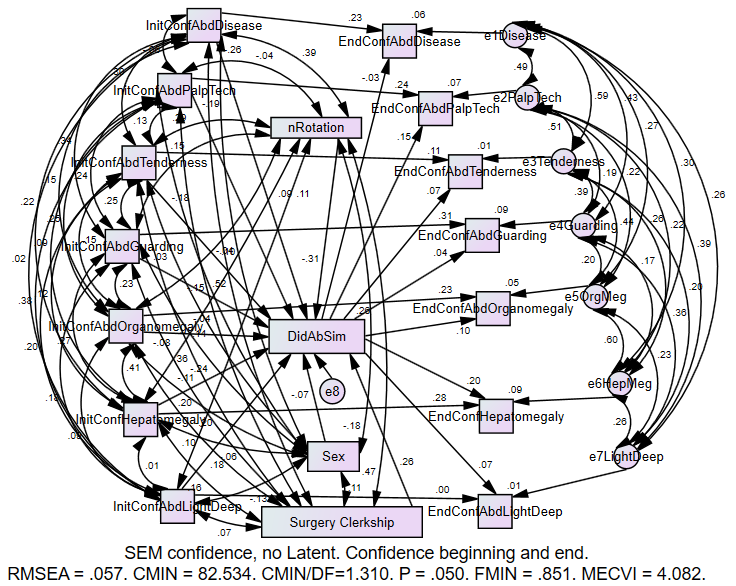


The intercorrelations between every pair of items, at each time, are represented and it can be seen that they are more strongly intercorrelated at the end than the beginning, perhaps because the students’ understanding became more coherent; or maybe they rushed through the questionnaire the second time they saw it (questionnaire completion time was not measured). The hypothesized relation with AbSim would be present as a numerical pattern in a triangle: initial confidence would negatively predict doing AbSim, initial confidence would predict final confidence, and doing AbSim would be positively associated with final confidence.

The unstandardized parameter estimates of interest are in the following table. Confidence is measured on a 4-point ordinal scale, and AbSim is a dichotomous variable. The expected “negative, positive, positive” relations are present for 4 of the seven variables, and the non-conforming relation is near 0 for two of the others. The “ability to identify right hepatic lobe” is an unclear concept and was not addressed by the tutorial. Yet it is the only one that the AbSim tutorial strongly affected.

| Confidence about: | Pre-Confid 🡪 AbSim | PreConf 🡪 PostConf | AbSim 🡪 PostConf |
| --- | --- | --- | --- |
| identifying abdominal disease states and their respective physical findings | -.228* | .215** | -.037 |
| using correct technique to palpate the abdomen | -.197 | .272** | .158+ |
| your ability to attribute abdominal tenderness to the right organ | -.153^+^ | .108 | .108 |
| your ability to recognize muscular guarding during the abdominal exam | .021 | .302*** | .048 |
| your ability to identify abdominal organomegaly | -.064 | .263** | .133 |
| your ability to identify right hepatic lobe enlargement | -.088 | .340*** | .302*** |
| your ability to use light and deep palpation | .156^+^ | .002 | .088 |

The next model with this set of students has a latent variable of all the individual confidence items, for the pre and for the post items. The parameters are standardized. The latent variable itself represents what is common among the items, so we do not include pairwise correlations among the error terms of the items. Thus we can look for the expected pattern in just one place, the triangle of initial confidence, final confidence, and AbSim. It is the expected pattern: the more confidence initially, the less likely to do AbSim (β = -0.61, p = .003) and the more confidence at the end (β = 0.69, p < .001); doing AbSim predicts more confidence at the end (β = 0.34, p = .013). This model does not explain all the relations in the model as well as the previous, because if there are stronger pairwise relations among some items than others, the model does not recognize the fact. In this model, those who had already done the surgery rotation were more likely to study with the AbSim tutorial, which is not consistent with the multivariate analyses presented in Appendix 6.


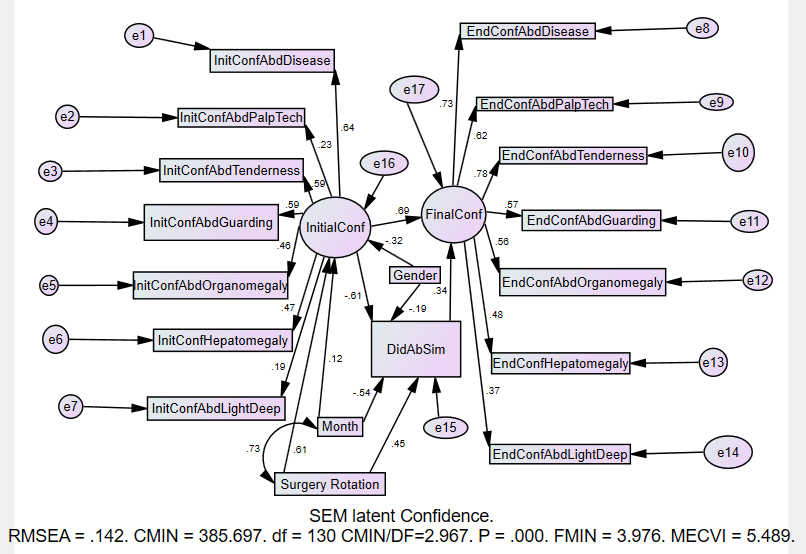


To address the non-uniformity of the intercorrelations among the items, we use three latent variables instead of one for the confidence items, in effect a factor analysis with three correlated factors. The factors are 1) identifying diseases, guarding, and tenderness, 2) palpation technique and distinguishing light from deep (the only one conceivably influenced by the tutorial’s training, but the students don’t know this), and 3) identifying organomegaly and the ill defined “right hepatic megaly”. Here is the standardized variable representation.


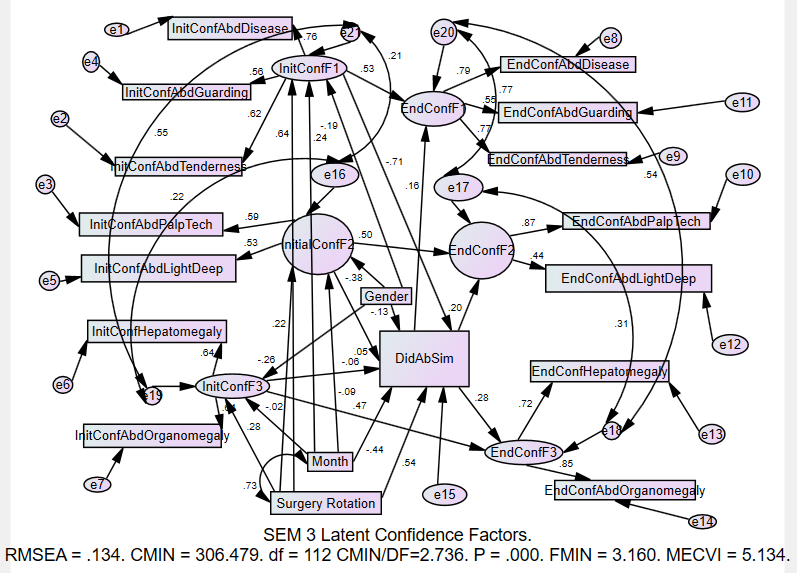


The expected pattern is seen for factors 1 and 3. The connections from initial confidence to AbSim differ, being strong for Factor 1 and nonsignificant for Factors 2 and 3. The connections to Final Confidence are essentially the same for each of the three factors.

| Factor 1. Identifying abnormalities. | To AbSim | To Final Confidence |
| --- | --- | --- |
| From Initial Confidence | -0.71 | 0.53 |
| From AbSim |  | 0.16 |

| Factor 2. Palpation technique. | To AbSim | To Final Confidence |
| --- | --- | --- |
| From Initial Confidence | 0.05 | 0.50 |
| From AbSim |  | 0.20 |

| Factor 3. Identifying megaly. | To AbSim | To Final Confidence |
| --- | --- | --- |
| From Initial Confidence | -0.06 | 0.47 |
| From AbSim |  | 0.28 |

Appendix 7b. The second set of structural equation models exclude the Abdominal Simulator Tutorial as a variable. Only the students who took the tutorial have any performance measures, so these are fit to the data of 68 students who did the tutorial and have all data.

Gender

Pre-FM Clerkship Experience (Int Med, Surg, and months of 3^rd^ year)

Pre-Tutorial Performance

Pre-Clerkship Confidence

Post-Tutorial Performance

Post-Clerkship Confidence

Here is its representation in the AMOS SEM program. The expected pattern is that each of the measures, confidence and performance (area covered and depth calibration), should have some stability from the beginning of the clerkship to the end of the clerkship (for confidence) or the end of the training (for performance); that initial confidence should have a relation to initial performance (if the students have any accurate self perception of their abilities), and that final confidence should reflect final performance. This can be envisioned as box with all 4 connections being positive. For simplicity, we use the average of the 7 confidence items (rather than the latent variable).


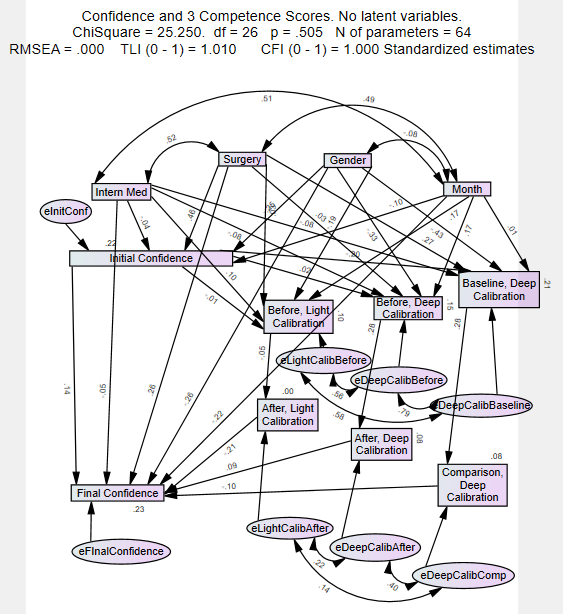


Initial confidence does not show the expected relation with initial performance. It is essentially unrelated to light calibration and to deep calibration, while it has a negative relation to the baseline measure. All measures show some stability, with the light calibration having the least. Deep calibration after training has a positive relation with final confidence, while the light calibration and the comparison measure had negative relations.

Next, we model the performance measures using a latent variable. We know that the deep calibration measure and the baseline/comparison measure will be tightly correlated, because both include the deep palpation, and so they will probably dominate the latent variable. We get no relation between initial confidence and pre-tutorial performance, some stability of each measure, and a negative relation between post tutorial performance and end of clerkship confidence.


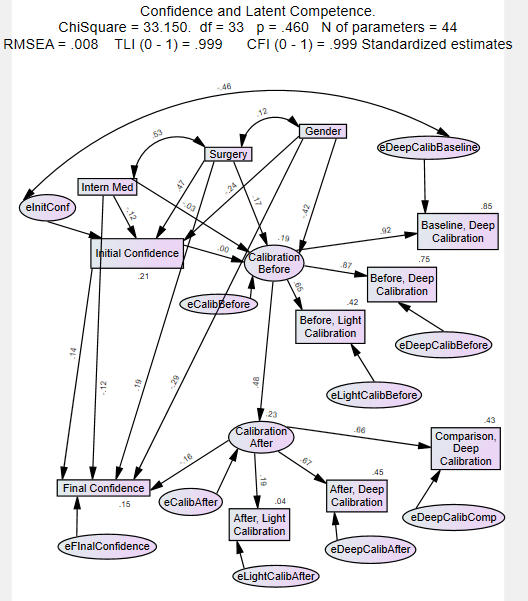


Because our license to AMOS expired, we stopped the explorations here. The multivariate analyses afforded by SEM modeling produced some larger relations than the multivariate analyses and the pairwise correlations revealed. These affirm the notion that the initial confidence negatively predicted doing the tutorial, while doing the tutorial positively affected the final confidence. This indirect effect would decrease the overall relation between initial and final confidence, and it casts doubt on the assumption that this training is good for the confidence of all students, but substitutes the possibility that the training is good as a remediation for students with low confidence. The SEM analyses addressing the relation between student confidence in abdominal palpation skill and their measured performance finds only very weak and somewhat contradictory relations.

References for supplementary appendices.

Eva, K. W., Cunnington, J. P., Reiter, H. I., Keane, D. R., & Norman, G. R. (2004). How can I know what I don't know? Poor self assessment in a well-defined domain. *Adv Health Sci Educ Theory Pract, 9*(3), 211-224. doi:10.1023/B:AHSE.0000038209.65714.d4

Eva, K. W., & Regehr, G. (2005). Self-assessment in the health professions: a reformulation and research agenda. *Acad Med, 80*(10 Suppl), S46-54. doi:10.1097/00001888-200510001-00015

McGaghie, W. C., Issenberg, S. B., Petrusa, E. R., & Scalese, R. J. (2010). A critical review of simulation-based medical education research: 2003-2009. *Med Educ, 44*(1), 50-63. doi:10.1111/j.1365-2923.2009.03547.x

Moore, D. E., Jr., Green, J. S., & Gallis, H. A. (2009). Achieving desired results and improved outcomes: integrating planning and assessment throughout learning activities. *J Contin Educ Health Prof, 29*(1), 1-15. doi:10.1002/chp.20001

Weinstein, N. D. (1980). Unrealistic Optimism About Future Life Events. *Journal of Personality and Social Psychology, 39*, 806-820.

1. This means that relative preference for studying with a simulator, individually or in a group, did not predict choosing to do it. Because there was essentially no relation between the preference ranks and other variables, we report no further analyses. [↑](#footnote-ref-1)
